# Classifying human vs. AI text with machine learning and explainable transformer models

**DOI:** 10.1038/s41598-025-27377-z

**Published:** 2025-12-08

**Authors:** Adven Masih, Bushra Afzal, Shamyla Firdoos, Jabar Mahmood, Aitizaz Ali, Mohamed Shabbir Abdulnabi, Daniel Musafiri Balungu

**Affiliations:** 1 Faculty of Computing and Information Technology, University of Sialkot, Daska Road, Sialkot, 51040 Punjab Pakistan; 2 State Key Laboratory of Blockchain and Data Security, School of Cyber Science and Technology, College of Computer Science and Technology, Zhejiang University, Hangzhou, 310007 Zhejiang China; 3 Hangzhou High-Tech Zone (Binjiang) Institute of Blockchain and Data Security, Hangzhou, Zhejiang China; 4 School of Technology (SOT), Asia Pacific University of Technology and Innovation (APU), Kuala Lumpur, 57000 Malaysia; 5 Department of Big Data Analytics and Video Analysis Methods, Ural Federal University, Yekaterinburg, 620002 Russia

**Keywords:** Large language models (LLMs), Recurrent deep learning, Transformer models, Text classification, AI generated text detection, Natural language processing (NLP), GPT-4, Human-Generated text, Engineering, Mathematics and computing

## Abstract

The rapid proliferation of AI-generated text from models such as ChatGPT-3.5 and ChatGPT-4 has raised critical challenges in verifying content authenticity and ensuring ethical use of language technologies. This study presents a comprehensive framework for distinguishing between human-written and GPT-generated text using a combination of machine learning, sequential deep learning, and transformer-based models. A balanced dataset of 20,000 samples was compiled, incorporating diverse linguistic and topical sources. Traditional algorithms and sequential architectures (LSTM, GRU, BiLSTM, BiGRU) were compared against advanced transformer models, including BERT, DistilBERT, ALBERT, and RoBERTa. Experimental findings revealed that RoBERTa achieved the highest performance (Accuracy = 96.1%), outperforming all baselines. Post-hoc temperature scaling (T = 1.476) improved calibration, while threshold tuning (t = 0.957) enhanced precision for high-stakes applications. McNemar’s test with Holm correction confirmed the statistical significance (*p* < 0.05) of RoBERTa’s superiority. Efficiency analysis showed optimal trade-offs between accuracy and latency, and 20% pruning demonstrated sustainability potential. Furthermore, LIME and SHAP explainability analyses highlighted linguistic distinctions between AI-generated and human-authored text, and fine-grained error evaluation confirmed model robustness across text lengths. In conclusion, RoBERTa emerges as a reliable, interpretable, and computationally efficient model for detecting AI-generated content.

## Introduction

The rapid advancement of large language models (LLMs), such as OpenAI’s ChatGPT and its successors, has transformed natural language processing (NLP), enabling applications ranging from creative content generation to automated code writing and essay composition^1–4^. Built on the transformer architecture with self-attention mechanisms, these models, including GPT-3.5 and GPT-4, excel at capturing long-range dependencies and producing human-like text^1,2,5^. However, the proliferation of AI-generated content raises significant concerns about accountability, authenticity, and potential misuse, particularly in online content moderation, academic integrity, and creative industries^6^. The ability to accurately distinguish between human-authored and AI-generated text is thus critical to mitigating risks such as misinformation, plagiarism, and erosion of trust in digital platforms^7^.

Prior research has explored various approaches to this challenge, including statistical language models, energy-based models like Boltzmann machines, and recurrent architectures such as LSTMs and GRUs^8–12^. While these methods offer insights, they often struggle with data sparsity or fail to capture the complex patterns of modern AI-generated text. Transformer-based models, such as BERT^13^ and its lightweight variant DistilBERT^14^, have demonstrated superior performance in NLP tasks by leveraging bidirectional context and pre-trained representations. Despite these advances, two key limitations persist: (1) existing datasets lack diversity in text types, particularly those generated by advanced models like GPT-4, which closely mimic human writing, and (2) models trained on earlier AI outputs (e.g., GPT-3.5) are less effective at detecting text from newer, more sophisticated models^15^.

This study addresses these gaps by developing a robust classification framework for differentiating between human-written and AI-generated text from GPT-3.5 and GPT-4. A balanced dataset of 20,000 samples was constructed by integrating multiple publicly available sources, ensuring both diversity and representativeness. Multiple modeling approaches were evaluated, spanning traditional machine learning classifiers, deep neural networks, and advanced transformer models. The results highlight RoBERTa as the strongest performer, achieving high accuracy with consistent reliability. To enhance transparency and trust, Explainable AI (XAI) techniques specifically LIME and SHAP,  were applied, which provided fine-grained insights into the linguistic features driving model predictions, to elucidate the model’s decision-making process by visualizing the impact of specific text features on predictions^16^. The primary objectives of this study are:

1. To construct a balanced and linguistically diverse dataset integrating human-written and GPT-generated texts from multiple open repositories, and to preprocess the data through normalization, tokenization, and embedding generation to ensure robust and unbiased model training.
2. To design, train, and compare a wide range of models including traditional machine learning algorithms (SVM, Random Forest, Logistic Regression), deep learning architectures (RNN, LSTM, GRU, BiLSTM, BiGRU), and transformer-based models (BERT, DistilBERT, ALBERT, RoBERTa, XLM-RoBERTa, DeRoBERTa) for accurate classification of AI-generated versus human-authored content.
3. To enhance model reliability, interpretability, and fairness through post-hoc calibration (temperature scaling), precision-oriented threshold tuning, and explainable AI techniques (LIME and SHAP), supported by statistical validation (McNemar’s test with Holm correction) and fine-grained error analysis across text-length categories.
4. To evaluate model efficiency by analyzing inference latency, throughput, and pruning-based compression, with a focus on assessing the model’s suitability and optimization for real-time deployment in practical environments.

The remainder of this article is structured as follows. The next section reviews the related literature on AI-generated text detection and identifies existing gaps. The following section outlines the methodology, including dataset construction, model design, and the integration of explainable AI and calibration techniques. Subsequent sections present comparative model performance, statistical validation, and fine-grained error analysis,  followed by the conclusion outlining key findings, limitations, and future directions. 

## Literature review

The proliferation of large language models like ChatGPT has heightened the need to distinguish AI-generated text from human-authored content, driven by concerns over authenticity, accountability, and potential misuse. Existing research provides valuable insights into detection methods but reveals critical limitations in dataset diversity and model adaptability to advanced AI outputs.

For example, Ippolito et al. demonstrated that even trained individuals struggle to differentiate AI-generated text from human-written content, underscoring the linguistic sophistication of LLMs^17^. This similarity raises concerns about misinformation and authenticity across domains. Similarly, Solaiman et al. (2019) highlighted the ethical risks of releasing models like GPT-2, noting their potential for generating misleading content or facilitating plagiarism, and stressed the need for responsible deployment and regulation^18^. Jawahar et al. (2020) provided a comprehensive survey of detection techniques, including statistical, feature-based, and deep learning approaches, emphasizing the complexity of identifying machine-generated text due to varying content types and model characteristics^19^.

Recent studies have explored machine learning and deep neural networks for text differentiation. Islam et al. (2023) evaluated 11 algorithms, including Support Vector Machines, K-Nearest Neighbors (KNN), and Logistic Regression, on a dataset of 10,000 text records from GPT-3.5 and human sources, achieving a peak accuracy of 77%^15^. A state of the art work employed advanced models like RoBERTa and T5 on the OpenGPT Text dataset (30,000 samples), attaining over 97% accuracy, highlighting the efficacy of transformer-based architectures^20^. In a domain-specific context. Liao et al. conducted a pioneer study to analyze medical texts, finding that human-written texts are more concrete and informative compared to ChatGPT’s fluent but less specific outputs^21^. On the other hand, Katib et al. proposed a Tunicate Swarm Algorithm with Long Short-Term Memory Recurrent Neural Networks (TSA-LSTMRNN), achieving accuracies of 93.17% and 93.83% on human and ChatGPT datasets, respectively, using feature extraction techniques like TF-IDF and word embeddings^22^. One of the recent works in this domain by Qazi et al. (2024) introduced the GPT Reddit Dataset (GRiD), benchmarking detection models on diverse Reddit-based context-prompt pairs, demonstrating improved performance in real-world settings^23^, whereas Prova compared XGB Classifier, SVM, and BERT, with BERT achieving 93% accuracy, reinforcing the strength of transformer models^5^.

Despite these advancements, two critical gaps persist. Firstly, datasets used in prior studies often lack diversity, failing to capture the varied text types produced by advanced models like GPT-4, which closely mimic human writing^15^. This limitation hampers model generalizability in real-world applications. Secondly, models trained primarily on earlier AI outputs, such as GPT-3.5, struggle to detect text from more sophisticated models like GPT-4, reducing detection accuracy. This study addresses these gaps by developing a binary classification model using a combined dataset of GPT-3.5 and GPT-4 outputs, alongside human-generated text, integrated with explainable AI techniques to enhance detection robustness, interpretability, and applicability across diverse AI-generated content.

## Methodology

**Fig. 1:**
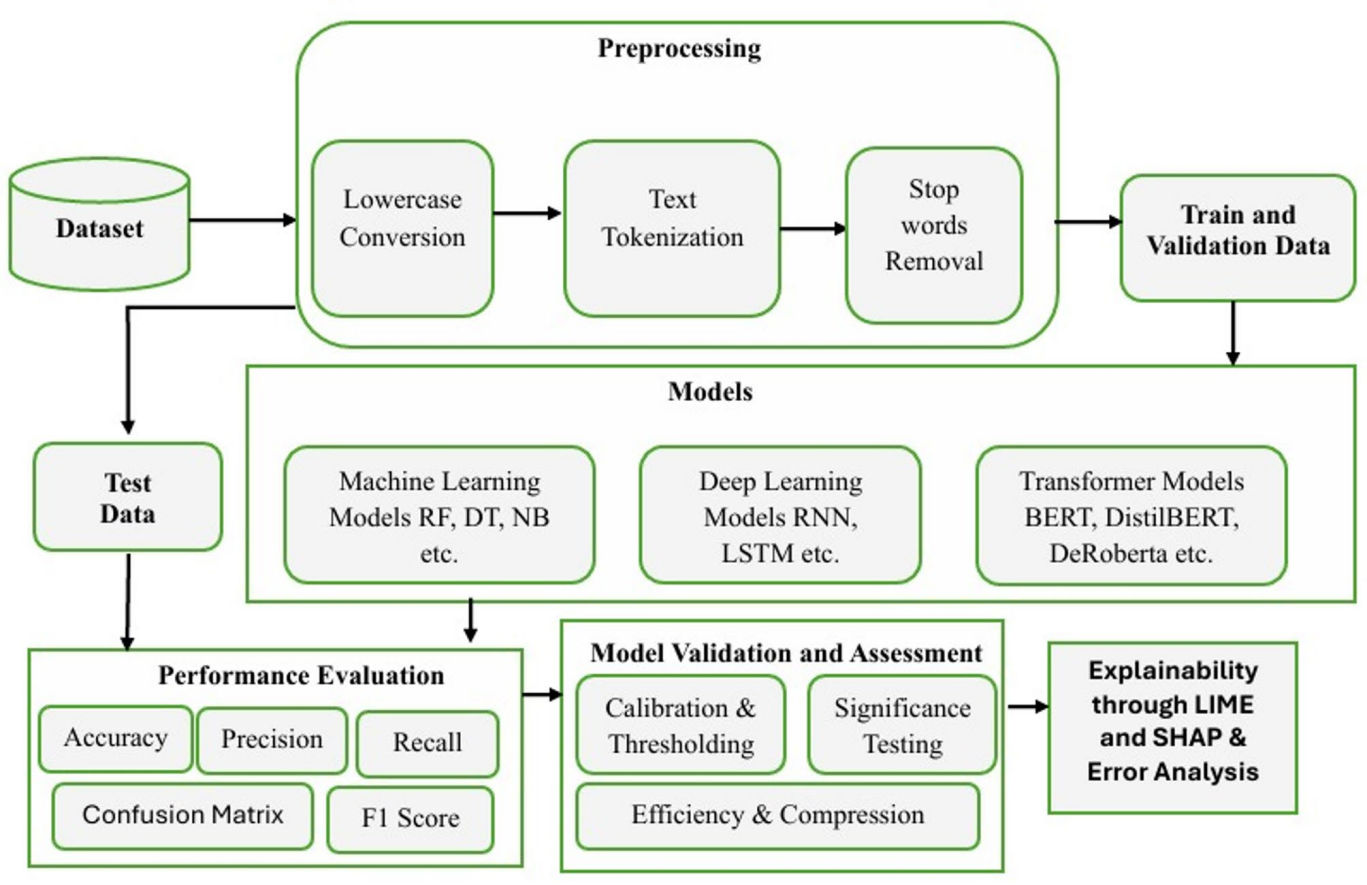
Methodology diagram of the study.

The methodology involved constructing a balanced dataset of 20,000 samples combining human-written and ChatGPT-generated texts from versions 3.5 and 4, followed by preprocessing through normalization, tokenization, and stratified splitting. A range of models including traditional ML, deep learning (LSTM, GRU), and transformer-based architectures (BERT, DistilBERT, RoBERTa, XLM-RoBERTa) were trained and fine-tuned under consistent settings. Model performance was evaluated using accuracy, precision, recall, F1-score, and confusion matrices, while temperature scaling improved calibration reliability and threshold tuning optimized decision confidence. McNemar’s test with Holm correction confirmed RoBERTa’s statistically significant superiority, and inference efficiency was analyzed through latency and throughput measures. To enhance interpretability and sustainability, unstructured pruning (20%) was performed, and explainability tools (LIME and SHAP) were applied, supported by fine-grained error analysis across text length categories to validate model robustness and reliability. The comprehensive methodology employed in this research is outlined in Fig. 1.

### Data collection

This study leverages a comprehensive dataset sourced from Kaggle, combining three distinct datasets to form a balanced sample of 20,000 instances for binary classification of human-authored and AI-generated text. The ChatGPT Classification Dataset comprises 10,000 samples, evenly split between 5,000 human-written texts and 5,000 ChatGPT-generated texts. The GPT Reddit Dataset (GRiD), utilized in the study “GPT-generated Text Detection: Benchmark Dataset and Tensor-based Detection Method,” includes 6,513 samples, with 5,145 human-authored texts and 1,368 texts generated by the GPT-3.5-turbo model. Additionally, all ChatGPT-4 Conversation dataset provides 5,000 GPT-4-generated texts. By integrating these sources, the final dataset consists of 10,000 human-written and 10,000 AI-generated instances (5,000 from GPT-3.5 and 5,000 from GPT-4), as shown in Table 1, ensuring a robust and diverse foundation for evaluating text classification models.

**Table 1:** Description of dataset.

Class	Labels	Public source	Count	References
0	Human	GRiD (GPT Reddit Dataset)	5000	^24^
1	AI	ChatGPT Classification Dataset (Kaggle)	5000	^25^
0	Human	ChatGPT Classification Dataset (Kaggle)	5000	^25^
1	AI	All ChatGPT-4 Conversations (Kaggle)	5000	^26^
Total			20,000	

The dataset is annotated for binary classification, with a label of 0 assigned to human-authored text and 1 assigned to AI-generated text from ChatGPT (including GPT-3.5 and GPT-4). Sample instances include human-written texts, labeled as 0, sourced from diverse contexts such as news articles and social media, and AI-generated texts, labeled as 1, produced by GPT-3.5 and GPT-4, reflecting their coherent and human-like characteristics as shown in Table 2. The dataset considered under this study are publicly available at https://github.com/shamylafirdoos/Gpt-vs-Human-Text-Classification.

**Table 2:** Representative samples of the dataset.

Data	Labels
NLP is a multidisciplinary field that draws from linguistics and computer science, particularly artificial intelligence	0
Of course each language has its own forms of ambiguity.	0
As formidable as the task of extracting the correct (literal) meaning from text can be, it is really only the first level of natural language processing.	0
The political stereotypes you mentioned in the 1994 Simpsons episode “Bart Gets an Elephant” are satirical portrayals of the two major political parties in the United States: the Democrats and the Republicans. Such stereotypes have developed and evolved over a long period of time, influenced by various historical events, cultural shifts, and political ideologies. The roots of these stereotypes can be traced back to the early years of the American republic.	1
The concept of cultural nostalgia was not unique to modern times. During the Roman Empire, the idea of a “Golden Age” was prevalent, and many Romans believed that earlier periods in Roman history were superior to their own time.	1
Yes, there are several sources that provide information on the population of the United States during the period between the signing of the Constitution in 1787 and the outbreak of the	1

### Exploratory data analysis (EDA)

To gain a better understanding of the dataset’s structure and content for the task of distinguishing Human-Generated from GPT-Generated text, several exploratory data analysis techniques were applied. These included statistical summaries, distribution analysis, and word frequency visualization.

**Fig. 2:**
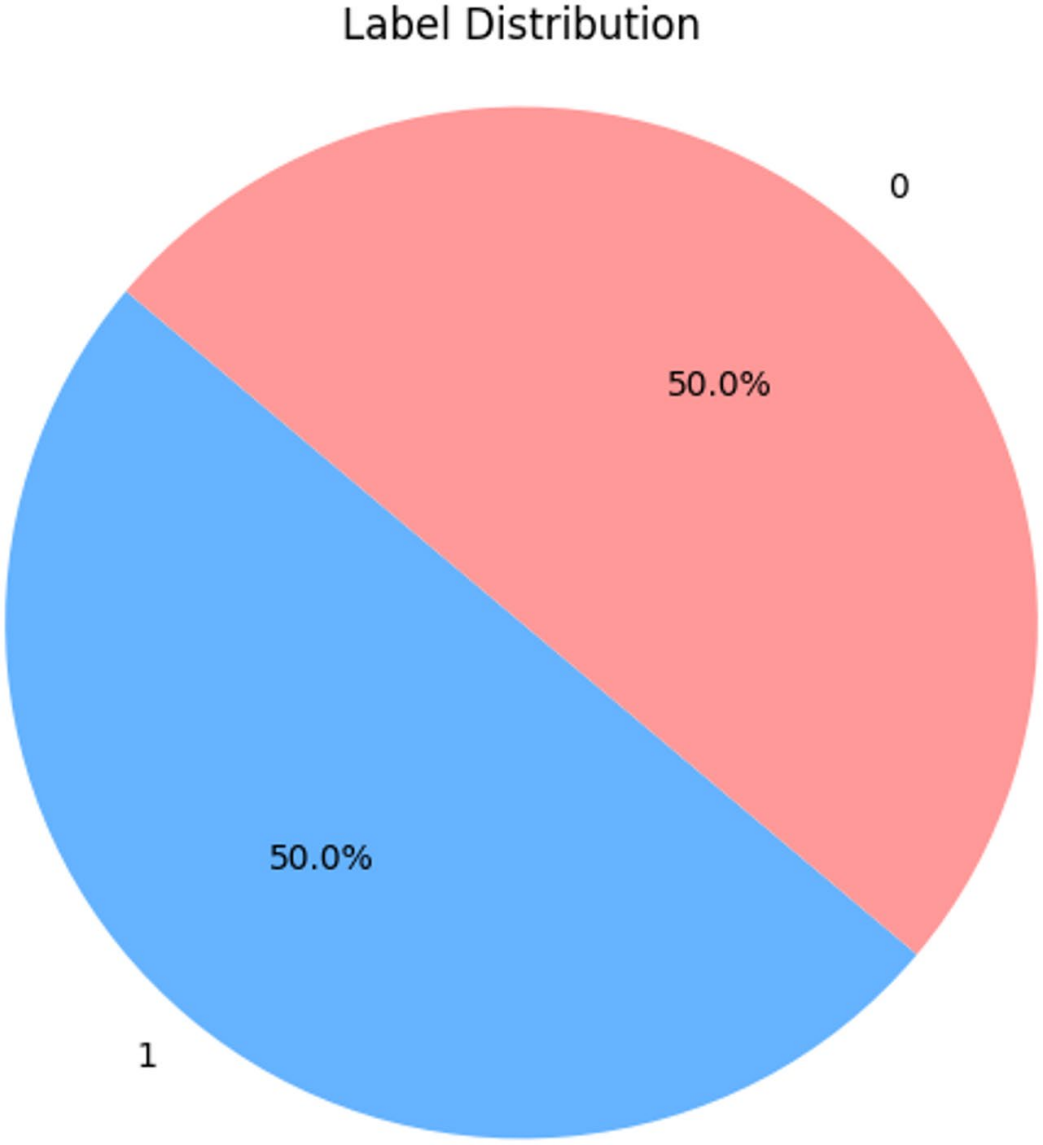
Label distribution of dataset.

Figure 2 shows that, the dataset is evenly balanced, with 50% of the samples labeled as Human-Generated (label 0) and 50% as GPT-Generated (label 1). This balance ensures that the model is not biased toward any specific class during training, which is crucial for reliable classification performance.

**Fig. 3:**
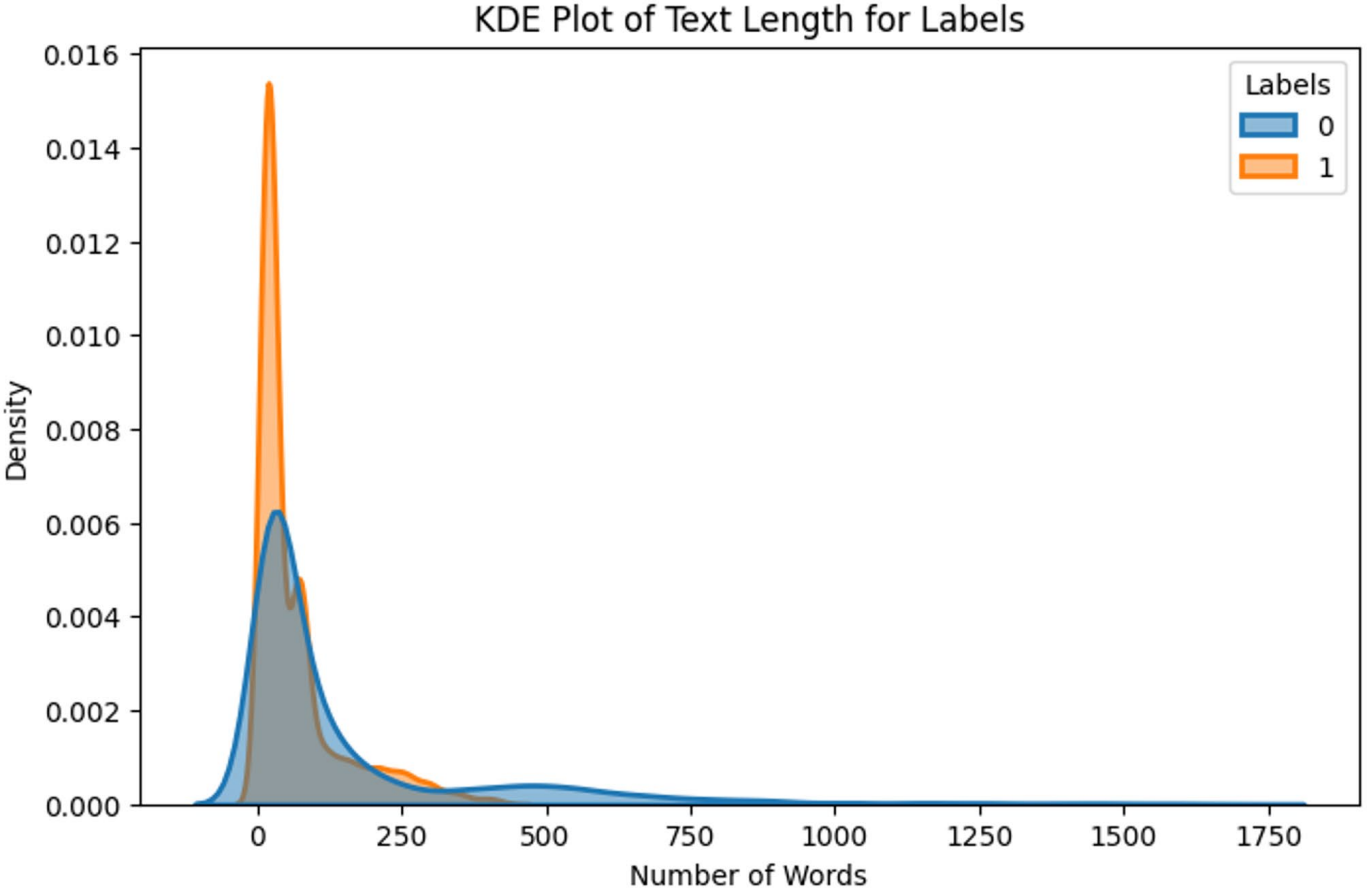
Text length distribution of dataset.

A Kernel Density Estimation (KDE) plot was generated to visualize the distribution of text lengths (in number of words) across both labels (0 and 1). As shown in Fig. 3, label 0 has a wider distribution with longer texts, whereas label 1 exhibits a steeper peak, indicating that texts are generally shorter. Most entries for both classes contain fewer than 250 words, with a sharp concentration under 100 words for label 1. This reflects a tendency for class 1 statements to be more concise, while class 0 statements are generally more elaborative.

**Table 3:** KDE observations.

Label	Avg. word count	Avg. word length	Avg. token count
0	150.22	5.03	150.22
1	66.68	5.32	66.68

Furthermore, dataset statistics presented in Table 3 support the KDE observations. For instance, Label 0 samples are, on average, more than twice as long as label 1 samples. Interestingly, while label 1 samples are shorter in word count, they tend to use slightly longer words on average. This suggests that GPT outputs may be more information-dense, possibly reflecting a formal or technical tone learned from its training corpus. To better understand the vocabulary associated with each class, word clouds were created.

**Fig. 4:**
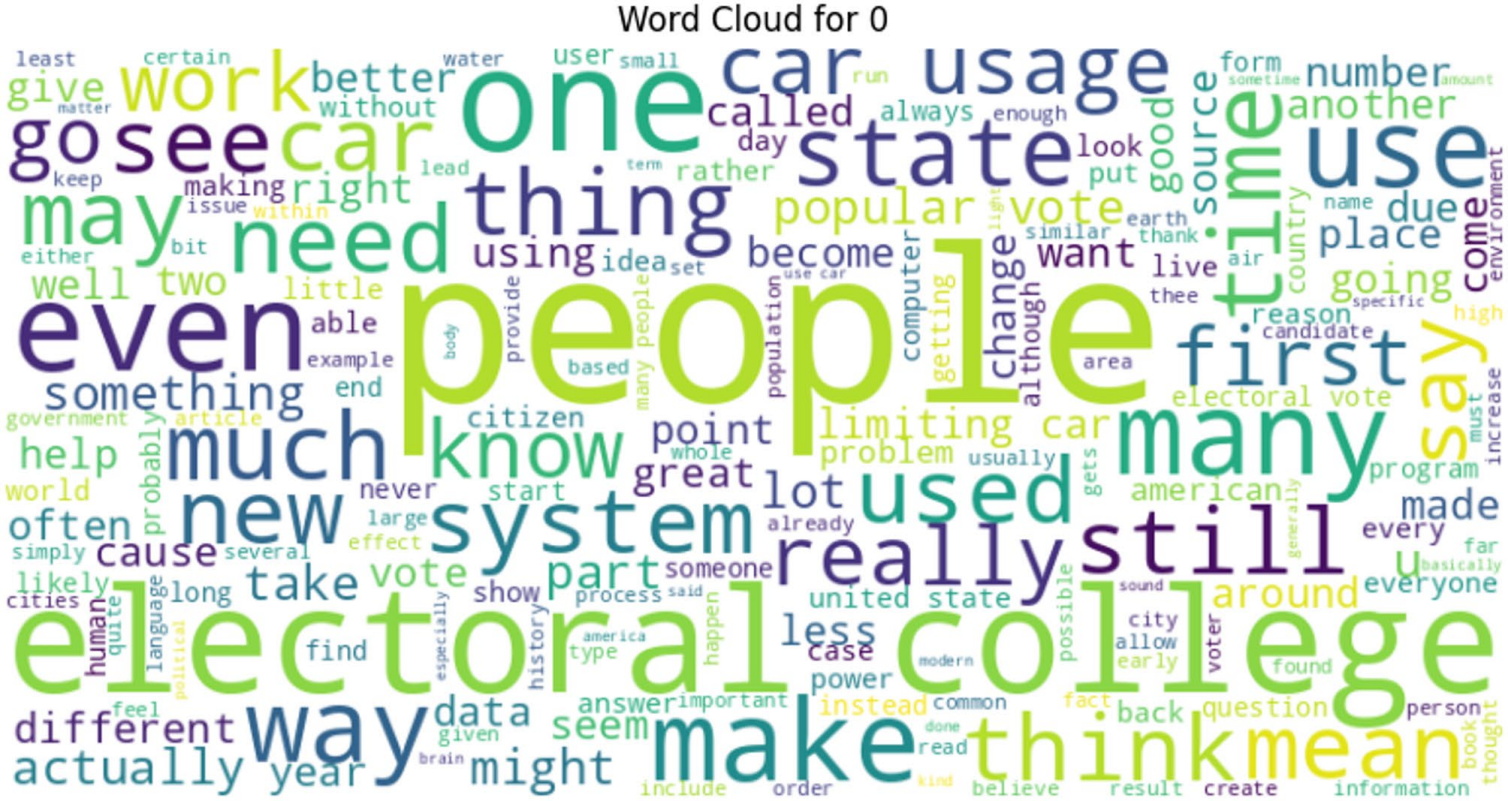
Label 0 word cloud.

**Fig. 5:**
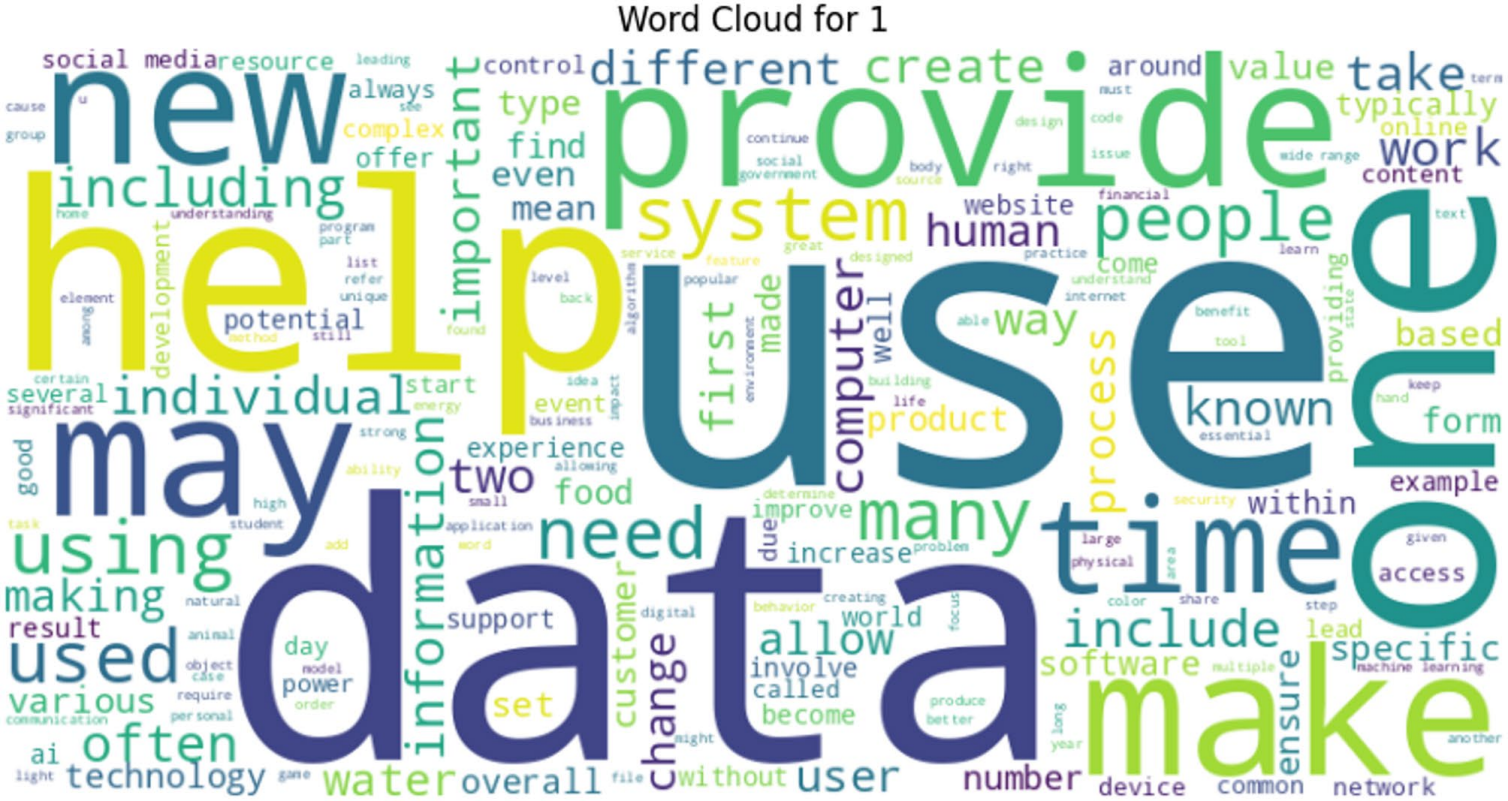
Label 1 word cloud.

Likewise, Word Cloud presented in Fig. 4 for Label 0 shows that common words under this class include people, electoral college, system, use, and even. It suggests that human-authored texts may focus more on societal, political, or opinion-based topics. Whereas, the Word Cloud presented in Fig. 5 reflects that data, provide, use, help, system, and information are the most frequency occurring terms under Label 1. The dominance of these words indicates that GPT-generated texts often reflect a formal, instructional, or factual style, consistent with the model’s typical response structure.

### Data preprocessing

To prepare the textual data for classification, several preprocessing steps were applied to clean and standardize the input:

- All text entries were converted to lowercase to maintain consistency and avoid treating the same word in different cases (e.g., “GPT” vs. “gpt”) as separate tokens. This normalization step is essential for reducing vocabulary size and improving embedding accuracy.
- The dataset was tokenized, which breaks down each sentence into individual word tokens based on whitespace. Tokenization facilitates further processing such as filtering and embedding generation.
- Common English stopwords were removed using NLTK’s predefined stopword list. Additionally, punctuation characters were excluded using Python’s string.punctuation. This step eliminates non-essential words and symbols that don’t typically contribute to the semantic understanding of the text.

### Embedding techniques

To effectively convert textual data into numerical form suitable for machine learning and sequential models, 3 distinct word embedding techniques namely Word2Vec, GloVe, and FastText were employed. These embeddings capture semantic and syntactic relationships between words by representing them as dense, low-dimensional vectors.

- Word2Vec, introduced by Mikolov et al.^27^, uses shallow neural networks to learn word representations based on their contextual co-occurrence in a large corpus. It provides two training architectures “Continuous Bag of Words (CBOW) and Skip-gram” which are effective at capturing semantic similarities. In the context of distinguishing between GPT and human-written text, it enables the model to recognize subtle linguistic patterns that may differ between AI-generated and natural human expression.
- GloVe (Global Vectors for Word Representation), developed by Pennington et al.^28^, constructs embeddings by analyzing global word-word co-occurrence statistics from a corpus. Unlike Word2Vec, which focuses on local context windows, GloVe incorporates broader statistical information, allowing for improved performance on tasks that require understanding global textual structure. This can be especially useful in identifying formal or repetitive structures common in GPT-generated content.
- FastText, developed by Facebook AI Research, extends Word2Vec by incorporating subword information. It represents words as bags of character n-grams^29^, which allows the model to generate embeddings for out-of-vocabulary or misspelled words i.e., something often seen in human writing. This feature enhances the classifier’s ability to detect informal or non-standard text patterns typically associated with human authorship, thus making FastText particularly robust for this binary classification task.

By transforming raw text into rich vector representations, these embedding techniques serve as a foundational layer for traditional classifiers and deep learning models. They enable the detection of fine-grained textual differences between GPT-generated and human-authored content, contributing significantly to model accuracy and generalization.

### Machine learning models

For initial experimentation, traditional machine learning models were adopted to classify text as GPT-generated or human-written. These models included SVM, RF, DT, LR, NB, and KNN. After preprocessing, the text was transformed into numerical vectors using embedding techniques like Word2Vec, GloVe, and FastText. These vector representations served as input features for the classifiers. SVM, known for its ability to handle high-dimensional data, provided competitive performance^30^. Ensemble models like Random Forest^31^ captured non-linear relationships effectively, while KNN classified text based on proximity in embedding space. These models established strong baselines for comparison with more complex deep neural networks and transformer-based architectures.

### Deep learning models

To better model the sequential nature of language, Sequence-to-Sequence Neural Models were implemented using Keras and TensorFlow. Recurrent Neural Networks^32^ were first applied but were limited by vanishing gradient issues. To address this, Long Short-Term Memory^11^ and Gated Recurrent Unit models were used due to their improved memory and ability to retain long-term dependencies^33^. Bidirectional versions like BiLSTM and BiGRU further enhanced context learning by processing input sequences in both forward and backward directions. The models received tokenized sequences embedded with pre-trained vectors, such as those from FastText.

### Transfer learning with transformer-base models

Furthermore, State-of-the-art transformer models were used to assess classification accuracy. Pre-trained models such as BERT, RoBERTa, DeRoberta, DistilBERT, ALBERT, multilingual BERT (mBERT), and XLM-RoBERTa were fine-tuned on the GPT vs. Human dataset. BERT uses a bidirectional transformer to understand context from both directions in text^34^. RoBERTa refines BERT’s training procedure by removing next sentence prediction and training with more data^35^. Lighter models like DistilBERT and ALBERT reduce computation time while retaining competitive performance^14^. Multilingual models (mBERT and XLM-R) were also evaluated to test cross-lingual effectiveness^36^.

### Reliability calibration and threshold optimization

To assess the trustworthiness of probability estimates, temperature scaling was applied as a post-hoc calibration technique. The optimal temperature (T = 1.476) was determined via validation set optimization to minimize Expected Calibration Error (ECE), which decreased from 0.4923 to a well-calibrated level after scaling. Reliability diagrams were generated before and after calibration to visualize probability alignment. Additionally, threshold tuning was performed to optimize decision boundaries for deployment. A precision-prioritized threshold (t = 0.957) was selected to achieve ≥ 90% precision, ensuring reliability in high-stakes classification contexts such as academic or legal text verification.

### Statistical significance testing

To confirm whether observed differences between transformer models were statistically meaningful, McNemar’s test with Holm correction for multiple comparisons was applied. Effect sizes (Cohen’s g) were computed to quantify performance differences between top-performing models (BERT, RoBERTa, XLM-RoBERTa).

### Statistical significance testing

To confirm whether observed differences between transformer models were statistically meaningful, McNemar’s test with Holm correction for multiple comparisons was applied. Effect sizes (Cohen’s g) were computed to quantify performance differences between top-performing models (BERT, RoBERTa, XLM-RoBERTa). These tests established that RoBERTa’s superiority was statistically significant (*p* < 0.05).

### Model efficiency and compression

Inference efficiency was evaluated using latency (seconds per prediction) and throughput (texts per second) metrics to assess trade-offs between performance and computational cost. Furthermore, a 20% global unstructured pruning experiment was conducted on RoBERTa to investigate the feasibility of model compression. Results demonstrated minimal degradation in validation accuracy, aligning with the sustainability objective of reducing computational footprint.

### Explainability and fine-grained error analysis

Explainability was achieved through Local Interpretable Model-Agnostic Explanations (LIME) and SHapley Additive exPlanations (SHAP) applied to RoBERTa predictions. LIME explains predictions by perturbing the input and observing the impact on the output, highlighting which features (words) were most influential^37^. SHAP, on the other hand, leverages cooperative game theory to assign each feature a contribution score, offering a consistent and theoretically grounded measure of feature importance.

A fine-grained error analysis was also incorporated to evaluate model robustness across text characteristics. Specifically, performance was assessed across text-length categories (very short, short, medium, long), revealing consistent F1-scores with minimal degradation (only a slight drop for medium-length inputs).

## Experiments and results

### Performance metrics

In evaluating the performance of machine learning, Recurrent Deep Learning, and transformer-based models for classifying GPT-generated versus human-written text, a comprehensive suite of performance metrics was employed to ensure robustness and practical applicability. These metrics include the confusion matrix, accuracy, precision, recall, and F1 score, each offering critical insight into various aspects of model behavior. The confusion matrix is particularly valuable as it outlines the distribution of true positives (TP), false positives (FP), true negatives (TN), and false negatives (FN). This breakdown enables detailed analysis of misclassification trends i.e., distinguishing whether the model tends to misclassify human-written content as AI-generated or vice versa. Such insights are crucial for refining model behavior in real-world applications, where subtle linguistic cues can cause confusion between classes.

Accuracy, defined in Eq. (1), measures the proportion of correctly predicted instances out of all predictions. While it provides a general sense of performance, it can be misleading in the presence of class imbalance such as when the dataset contains more GPT-generated samples than human-written ones. Therefore, accuracy must be interpreted in conjunction with other metrics.

1 $$\:Accuracy=\:\frac{TP+TN}{TP+TN+FP+FN}$$

Precision shown in Eq. (2), evaluates the correctness of the model’s positive predictions. In the context of this task, high precision indicates that when the model predicts a text as GPT-generated, it is usually correct. This helps reduce false alarms, ensuring that naturally written human content is not mistakenly flagged as AI-generated.

2 $$\:Precision=\:\frac{TP}{TP+FP}$$

Recall defined in Eq. (3), assesses the model’s ability to correctly identify all relevant instances of a class. A high recall means the model can effectively detect most GPT-generated content, minimizing the likelihood that such texts go unnoticed.

3 $$\:Recall=\:\frac{TP}{TP+FN}$$

The F1 score presented in Eq. (4), provides a harmonic mean of precision and recall, serving as a balanced metric particularly useful when both false positives and false negatives are costly. For example, in content moderation or academic integrity settings, misclassifying human work as AI-generated (or vice versa) can have significant consequences. A high F1 score thus indicates the model’s strong overall ability to make accurate and reliable distinctions between the two text types.

4 $$\:F1\:Score=\:\frac{2\times\:Precision\:\times\:Recall}{Precision+Recall}$$

Collectively, these metrics offer a well-rounded evaluation framework. They enable not only assessment but also iterative refinement of models, ensuring that the system accurately distinguishes between GPT-generated and human-written text with minimal risk of misclassification.

### Experimental settings

All experiments were conducted using Kaggle’s cloud-based platform, which provides access to powerful computing resources including free GPUs. The environment supported Python 3 with libraries such as Scikit-learn, TensorFlow, Keras, PyTorch, and HuggingFace Transformers. The dataset was uploaded and processed directly within Kaggle Notebooks. Pre-trained embeddings (e.g., FastText) and transformer models (e.g., BERT, RoBERTa) were loaded from external sources or integrated via HuggingFace. Model training, evaluation, and visualization were performed end-to-end within this environment, ensuring a reproducible and scalable experimental setup.

In this study, various hyperparameters were carefully selected and tuned for machine learning, recurrent deep learning and transformer models to ensure optimal performance. Each model was fine-tuned using carefully selected hyperparameters to optimize performance. The detailed explanation regarding hyperparameters such as batch size, Optimizer, sequence length, dropout rate, learning rate, number of epochs etc., is provided in Table 4. some were varied according to models used.

**Table 4:** Hyperparameters for used models.

Models	Hyperparameters with values
Machine Learning Models	Embedding Dimension = 100, 200, 300, Random Seed = 7, 42, 123
Deep Learning Models	Learning rate = 1e − 3, Batch Size = 32, Max_Length = 200, Optimizer = Adam, Epoch = 30, Early Stopping Patience = 3, loss function = CrossEntropyLoss, Embedding Dimension = 100, 200, 300, Random Seed = 7, 42, 123
Transfer Learning Models	Learning rate = 3e − 5, Epoch = 3, 4, 5, Optimizer = AdamW, Max_Length = 256, Batch Size = 32, loss function = CrossEntropyLoss, Gradient Clipping = 1.0, Dropout Rate = 0.1

### Performance evaluation

To assess the effectiveness of various models in distinguishing between Human-Generated and GPT-Generated text, extensive experiments were conducted using traditional machine learning models, deep learning architectures, and state-of-the-art transformer-based models. The evaluation metrics included confusion matrix, Accuracy, Precision, Recall, and F1 Score.

Among classical algorithms shown in Table 5, RF consistently achieved higher accuracy across embeddings, with Word2Vec-based features yielding up to 0.788 accuracy. The performance of Logistic Regression was equally strong, especially with Word2Vec (0.783) and FastText (0.796), while SVM achieved competitive results with FastText (0.794). Naïve Bayes and Decision Tree models showed relatively lower performance, highlighting their limitations in capturing complex semantic patterns. Overall, the ML models achieved superior performance when integrated with FastText embeddings compared to Word2Vec and GloVe, highlighting FastText’s effectiveness in capturing contextual and subword-level information.

**Table 5:** Performance evaluation of machine learning models across different word embeddings.

Model	Dim	Seed	Word2Vec (Acc/*P*/*R*/F1)	GloVe (Acc/*P*/*R*/F1)	FastText (Acc/*P*/*R*/F1)
SVM	100	7	0.749/0.752/0.749/0.749	0.739/0.739/0.739/0.739	0.785/0.785/0.785/0.785
		42	0.746/0.749/0.746/0.746	0.738/0.739/0.738/0.738	0.783/0.783/0.783/0.783
		123	0.752/0.755/0.752/0.751	0.732/0.732/0.732/0.732	0.776/0.777/0.776/0.776
	200	7	0.744/0.748/0.744/0.744	0.744/0.744/0.744/0.744	0.789/0.789/0.789/0.789
		42	0.741/0.744/0.741/0.740	0.742/0.742/0.742/0.742	0.790/0.790/0.790/0.790
		123	0.745/0.748/0.745/0.744	0.744/0.744/0.744/0.744	0.787/0.788/0.787/0.787
	300	7	0.745/0.748/0.745/0.744	0.755/0.755/0.755/0.755	0.788/0.788/0.788/0.788
		42	0.747/0.752/0.747/0.747	0.754/0.755/0.754/0.754	0.794/0.794/0.794/0.794
		123	0.746/0.750/0.746/0.746	0.754/0.755/0.754/0.754	0.788/0.788/0.788/0.788
Naive Bayes	100	7	0.675/0.675/0.675/0.675	0.654/0.659/0.654/0.650	0.676/0.680/0.676/0.674
		42	0.675/0.675/0.675/0.675	0.643/0.646/0.643/0.639	0.677/0.678/0.677/0.676
		123	0.674/0.674/0.674/0.674	0.653/0.660/0.653/0.651	0.682/0.685/0.682/0.681
	200	7	0.680/0.680/0.680/0.680	0.642/0.650/0.642/0.637	0.667/0.671/0.667/0.665
		42	0.680/0.681/0.680/0.680	0.631/0.635/0.631/0.625	0.662/0.663/0.662/0.660
		123	0.680/0.680/0.680/0.680	0.641/0.651/0.641/0.637	0.664/0.668/0.664/0.662
	300	7	0.681/0.681/0.681/0.681	0.637/0.644/0.637/0.633	0.667/0.671/0.667/0.665
		42	0.682/0.683/0.682/0.682	0.631/0.635/0.631/0.625	0.660/0.661/0.660/0.658
		123	0.680/0.681/0.680/0.680	0.631/0.635/0.631/0.625	0.667/0.671/0.667/0.665
Logistic Reg.	100	7	0.756/0.757/0.756/0.756	0.736/0.736/0.736/0.736	0.776/0.776/0.776/0.776
		42	0.754/0.754/0.754/0.754	0.735/0.736/0.735/0.735	0.786/0.786/0.786/0.786
		123	0.753/0.754/0.753/0.753	0.735/0.736/0.735/0.735	0.777/0.777/0.777/0.777
	200	7	0.764/0.765/0.764/0.764	0.743/0.743/0.743/0.743	0.782/0.782/0.782/0.782
		42	0.763/0.764/0.763/0.763	0.743/0.743/0.743/0.743	0.788/0.788/0.788/0.788
		123	0.764/0.765/0.764/0.764	0.744/0.744/0.744/0.744	0.785/0.785/0.785/0.785
	300	7	0.768/0.769/0.768/0.768	0.758/0.758/0.758/0.758	0.796/0.796/0.796/0.796
		42	0.783/0.784/0.783/0.783	0.751/0.751/0.751/0.751	0.786/0.786/0.786/0.786
		123	0.775/0.775/0.775/0.775	0.751/0.751/0.751/0.751	0.796/0.796/0.796/0.796
Random Forest	100	7	0.777/0.779/0.777/0.776	0.759/0.765/0.759/0.757	0.800/0.805/0.800/0.799
		42	0.776/0.779/0.776/0.776	0.761/0.771/0.761/0.759	**0.806/0.810/0.806/0.805**
		123	0.778/0.780/0.778/0.778	0.758/0.762/0.758/0.757	0.799/0.802/0.799/0.798
	200	7	0.782/0.783/0.782/0.782	0.761/0.768/0.761/0.759	0.798/0.803/0.798/0.797
		42	0.788/0.790/0.788/0.788	0.763/0.773/0.763/0.762	0.802/0.809/0.802/0.801
		123	0.784/0.786/0.784/0.784	0.769/0.773/0.769/0.768	0.801/0.804/0.801/0.800
	300	7	0.778/0.779/0.778/0.778	0.757/0.765/0.757/0.756	0.802/0.807/0.802/0.802
		42	0.780/0.782/0.780/0.779	0.760/0.773/0.760/0.758	0.798/0.803/0.798/0.797
		123	0.775/0.776/0.775/0.775	0.760/0.773/0.760/0.758	0.802/0.807/0.802/0.802
Decision Tree	100	7	0.689/0.689/0.689/0.689	0.657/0.657/0.657/0.657	0.716/0.716/0.716/0.716
		42	0.689/0.689/0.689/0.689	0.649/0.650/0.649/0.649	0.699/0.700/0.699/0.699
		123	0.689/0.689/0.689/0.689	0.664/0.664/0.664/0.664	0.698/0.698/0.698/0.698
	200	7	0.696/0.696/0.696/0.696	0.655/0.655/0.655/0.655	0.693/0.693/0.693/0.693
		42	0.697/0.697/0.697/0.697	0.653/0.653/0.653/0.653	0.683/0.684/0.683/0.683
		123	0.696/0.696/0.696/0.696	0.670/0.670/0.670/0.670	0.680/0.680/0.680/0.680
	300	7	0.687/0.687/0.687/0.687	0.661/0.661/0.661/0.661	0.683/0.683/0.683/0.683
		42	0.687/0.687/0.687/0.687	0.654/0.654/0.654/0.654	0.688/0.688/0.688/0.688
		123	0.689/0.689/0.689/0.689	0.654/0.654/0.654/0.654	0.683/0.683/0.683/0.683
KNN	100	7	0.757/0.757/0.757/0.757	0.747/0.755/0.747/0.745	0.776/0.788/0.776/0.774
		42	0.755/0.756/0.755/0.755	0.753/0.759/0.753/0.751	0.787/0.794/0.787/0.785
		123	0.755/0.755/0.755/0.755	0.739/0.748/0.739/0.737	0.769/0.782/0.769/0.766
	200	7	0.757/0.757/0.757/0.757	0.741/0.752/0.741/0.738	0.766/0.779/0.766/0.763
		42	0.756/0.756/0.756/0.756	0.752/0.761/0.752/0.749	0.788/0.797/0.788/0.785
		123	0.758/0.758/0.758/0.758	0.744/0.744/0.744/0.744	0.769/0.782/0.769/0.766
	300	7	0.757/0.758/0.757/0.757	0.744/0.756/0.744/0.741	0.769/0.783/0.769/0.766
		42	0.756/0.756/0.756/0.756	0.747/0.757/0.747/0.744	0.781/0.792/0.781/0.778
		123	0.757/0.757/0.757/0.757	0.747/0.757/0.747/0.744	0.769/0.782/0.769/0.766

Bold values indicate the best performance for each metric.

Recurrent Deep Learning approaches demonstrated (Table 6) notable improvements over traditional ML models. LSTM and GRU architectures, along with their bidirectional variants, consistently outperformed simple RNNs. The best performance was observed with BiLSTM (Seed = 123, Dim = 200) and BiGRU (Seed = 123, Dim = 200), achieving accuracies of 0.8457 and 0.8467, respectively. These models effectively captured sequential dependencies and contextual information, contributing to superior recall and F1-scores. While RNNs showed stable performance, their results were generally lower compared to LSTM and GRU families, confirming the importance of gated mechanisms in handling long-term dependencies.

**Table 6:** Performance evaluation of recurrent deep learning models across different word embeddings.

Model	Seed	Dim	W2Vec (Acc/Prec/Rec/F1)	GloVe (Acc/Prec/Rec/F1)	FastText (Acc/Prec/Rec/F1)
RNN	42	100	0.7923/0.7649/0.8385/0.8000	0.7357/0.7208/0.7611/0.7404	0.7913/0.8007/0.7705/0.7853
BiRNN	42	100	0.7940/0.7960/0.7853/0.7907	0.7627/0.7706/0.7416/0.7558	0.8017/0.8189/0.7699/0.7936
RNN	42	200	0.7933/0.7974/0.7813/0.7893	0.7423/0.7711/0.6824/0.7240	0.7727/0.7952/0.7288/0.7605
BiRNN	42	200	0.7687/0.8438/0.6541/0.7369	0.7473/0.7716/0.6958/0.7318	0.7870/0.8071/0.7490/0.7770
RNN	7	200	0.7660/0.7620/0.7672/0.7646	0.7543/0.7847/0.7063/0.7435	0.7773/0.7821/0.7738/0.7779
BiRNN	7	200	0.7957/0.7893/0.8015/0.7953	0.7633/0.7574/0.7804/0.7687	0.7757/0.7578/0.8155/0.7856
RNN	7	100	0.7743/0.8228/0.6938/0.7528	0.7620/0.7270/0.8452/0.7817	0.7673/0.7596/0.7877/0.7734
BiRNN	7	100	0.7997/0.8290/0.7503/0.7877	0.7647/0.7627/0.7738/0.7682	0.7767/0.7730/0.7884/0.7806
RNN	123	100	0.7753/0.7391/0.8445/0.7883	0.7357/0.7393/0.7436/0.7414	0.7770/0.7518/0.8398/0.7933
BiRNN	123	100	0.7937/0.7564/0.8607/0.8052	0.7547/0.7779/0.7260/0.7510	0.7830/0.8167/0.7404/0.7767
RNN	123	200	0.7677/0.7369/0.8257/0.7788	0.7410/0.7554/0.7273/0.7411	0.7833/0.7675/0.8247/0.7951
BiRNN	123	200	0.7800/0.7671/0.7981/0.7823	0.7360/0.7557/0.7122/0.7333	0.7747/0.7931/0.7547/0.7735
LSTM	42	100	0.8337/0.8817/0.7672/0.8204	0.7983/0.8048/0.7826/0.7936	0.8330/0.8500/0.8048/0.8268
BiLSTM	42	100	0.8287/0.8517/0.7921/0.8208	0.8123/0.8327/0.7773/0.8040	0.8227/0.8437/0.7880/0.8149
LSTM	42	200	0.8430/0.8602/0.8156/0.8373	0.8000/0.8120/0.7759/0.7935	0.8430/0.8675/0.8062/0.8357
BiLSTM	42	200	0.8367/0.8537/0.8089/0.8307	0.8147/0.8341/0.7813/0.8068	0.8400/0.8331/0.8466/0.8398
LSTM	7	200	0.8353/0.8459/0.8163/0.8308	0.8203/0.8307/0.8082/0.8193	0.8487/0.8485/0.8519/0.8502
BiLSTM	7	200	0.8410/0.8388/0.8405/0.8397	0.8127/0.8162/0.8108/0.8135	**0.8517/0.8362/0.8776/0.8564**
LSTM	7	100	0.8523/0.8629/0.8345/0.8484	0.8117/0.8171/0.8069/0.8120	0.8317/0.8368/0.8274/0.8321
BiLSTM	7	100	0.8510/0.8345/0.8721/0.8529	0.8107/0.8069/0.8208/0.8138	0.8270/0.8243/0.8347/0.8294
LSTM	123	100	0.8273/0.8159/0.8412/0.8284	0.8007/0.8519/0.7371/0.7903	0.8307/0.8649/0.7914/0.8265
BiLSTM	123	100	0.8417/0.8422/0.8371/0.8397	0.8270/0.8681/0.7789/0.8211	0.8220/0.8682/0.7672/0.8146
LSTM	123	200	0.8407/0.8356/0.8445/0.8400	0.8237/0.8618/0.7789/0.8183	0.8303/0.8597/0.7973/0.8273
BiLSTM	123	200	0.8457/0.8315/0.8634/0.8471	0.8333/0.8414/0.8293/0.8353	0.8290/0.8878/0.7606/0.8193
GRU	42	100	0.8337/0.8669/0.7847/0.8237	0.8267/0.8199/0.8331/0.8264	0.8243/0.8761/0.7517/0.8091
BiGRU	42	100	0.8373/0.8590/0.8035/0.8303	0.8223/0.8120/0.8345/0.8231	0.8373/0.8626/0.7988/0.8295
GRU	42	200	0.8387/0.8584/0.8075/0.8322	0.7957/0.8017/0.7806/0.7910	0.8453/0.8539/0.8297/0.8416
BiGRU	42	200	0.8373/0.8363/0.8351/0.8357	0.8243/0.8393/0.7981/0.8182	0.8380/0.8613/0.8022/0.8307
GRU	7	200	0.8427/0.8468/0.8331/0.8399	0.8247/0.8367/0.8102/0.8233	0.8453/0.8368/0.8611/0.8488
BiGRU	7	200	0.8403/0.8694/0.7974/0.8319	0.8103/0.7842/0.8604/0.8206	0.8300/0.8432/0.8142/0.8284
GRU	7	100	0.8397/0.8473/0.8250/0.8360	0.8177/0.8040/0.8439/0.8235	0.8410/0.8282/0.8638/0.8456
BiGRU	7	100	0.8363/0.8491/0.8143/0.8313	0.8250/0.8305/0.8201/0.8253	0.8350/0.8106/0.8776/0.8428
GRU	123	200	0.8360/0.8309/0.8398/0.8353	0.8023/0.8188/0.7861/0.8021	0.8270/0.8571/0.7927/0.8236
BiGRU	123	200	0.8467/0.8543/0.8324/0.8432	0.7980/0.8547/0.7273/0.7859	0.8180/0.8638/0.7632/0.8104
GRU	123	100	0.8350/0.8228/0.8499/0.8361	0.8020/0.8539/0.7377/0.7916	0.8153/0.8635/0.7574/0.8070
BiGRU	123	100	0.8337/0.8517/0.8042/0.8273	0.8133/0.8646/0.7515/0.8041	0.8240/0.8824/0.7554/0.8140

Bold values indicate the best performance for each metric.

In contrast, the performance of Transformer-based models shown in Table 7 demonstrates a significant superiority over both classical machine learning and recurrent deep learning baselines, underscoring their strong capability in capturing complex contextual representations. For example, BERT achieved the highest overall accuracy of 0.9637 with an epoch value of 3, with balanced precision, recall, and F1-scores, indicating strong generalization. RoBERTa, mBERT and DeRoBERTa also delivered competitive results, with accuracies of 0.9617, 0.9530 and 0.9480, respectively, while ALBERT maintained slightly lower but stable performance. The results demonstrate that transfer learning with pre-trained transformer architectures provides substantial improvements over traditional embeddings and models by leveraging large-scale contextual knowledge.

**Table 7:** Performance evaluation of transfer learning models under different epoch values.

Model	Epoch	Accuracy	Precision	Recall	F1 Score
mBERT	3	0.9447	0.9472	0.9447	0.9446
	4	0.9530	0.9541	0.9530	0.9530
	5	0.9393	0.9428	0.9393	0.9392
BERT	3	**0.9637**	**0.9643**	**0.9637**	**0.9637**
	4	0.9597	0.9601	0.9597	0.9597
	5	0.9267	0.9336	0.9267	0.9264
DeRoBERTa	3	0.9457	0.9495	0.9457	0.9455
	4	0.9480	0.9519	0.9480	0.9479
	5	0.9300	0.9373	0.9300	0.9297
ALBERT	3	0.9383	0.9427	0.9383	0.9382
	4	0.9533	0.9543	0.9533	0.9533
	5	0.9503	0.9513	0.9503	0.9503
XLM-RoBERTa	3	0.9390	0.9435	0.9390	0.9388
	4	0.9587	0.9609	0.9587	0.9586
	5	0.9197	0.9288	0.9197	0.9192
DistilBERT	3	0.9603	0.9604	0.9603	0.9603
	4	0.9583	0.9587	0.9583	0.9583
	5	0.9497	0.9514	0.9497	0.9496
RoBERTa	3	0.9617	0.9622	0.9617	0.9617
	4	0.9537	0.9557	0.9537	0.9536
	5	0.9130	0.9238	0.9130	0.9124

Bold values indicate the best performance for each metric

Additionally, the study reports the performance of transfer learning models with 95% confidence intervals (CIs) computed over three random seeds (7, 42, and 123) for all key metrics, and further assess statistical significance and calibration reliability. Table 8 summarizes the results at epoch 3, identified in Table 7 as the optimal convergence point for most models. RoBERTa achieved the highest accuracy (0.961 ± 0.004) and F1-score (0.962 ± 0.004), followed by XLM-RoBERTa and BERT, while DeBERTa attained the best recall (0.991 ± 0.007) at the expense of precision, indicating a recall–accuracy trade-off. Paired McNemar tests confirmed the statistical significance of differences between BERT and the top-performing models. Calibration analysis further validated reliability, with RoBERTa exhibiting the lowest Brier score (0.034 ± 0.003) and stable ECE values across models. In terms of efficiency, DistilBERT required the least GPU time (0.862 h), highlighting its resource-friendliness despite slightly lower accuracy.

**Table 8:** Performance of transfer learning models with 95% confidence intervals (CIs) trained with 3 epoch.

Model	Accuracy (± CI)	Precision (± CI)	Recall (± CI)	F1 (± CI)	Brier (± CI)	ECE (± CI)	GPU Hours
BERT	0.950 ± 0.004	0.923 ± 0.007	0.983 ± 0.004	0.952 ± 0.004	0.034 ± 0.004	0.492 ± 0.011	1.725
DistilBERT	0.944 ± 0.009	0.923 ± 0.031	0.970 ± 0.023	0.946 ± 0.008	0.049 ± 0.009	0.489 ± 0.002	0.862
RoBERTa	**0.961 ± 0.004**	**0.945 ± 0.007**	**0.979 ± 0.004**	**0.962 ± 0.004**	**0.034 ± 0.003**	**0.492 ± 0.010**	1.717
ALBERT	0.946 ± 0.011	0.946 ± 0.042	0.948 ± 0.040	0.946 ± 0.010	0.047 ± 0.008	0.490 ± 0.004	1.797
mBERT	0.942 ± 0.008	0.911 ± 0.017	0.981 ± 0.006	0.945 ± 0.006	0.052 ± 0.008	0.490 ± 0.001	1.867
XLM-RoBERTa	0.956 ± 0.004	0.933 ± 0.007	0.982 ± 0.004	0.957 ± 0.004	0.039 ± 0.004	0.490 ± 0.010	1.781
DeBERTa	0.942 ± 0.031	0.905 ± 0.053	0.991 ± 0.007	0.945 ± 0.027	0.054 ± 0.032	0.494 ± 0.004	0.632

Bold values indicate the best performance for each metric

Furthermore, to evaluate the generalization capacity of RoBERTa, its classification performance was compared across three dataset versions: the original, a 5–10% human-edited, and a 30–40% human-edited version. This experiment aimed to examine the model’s robustness and brittleness under varying levels of realistic human post-editing. The results (Table 9) indicate that RoBERTa maintained strong performance on both edited datasets. For the 5–10% human-edited data, accuracy (0.951 ± 0.014) and F1 score (0.953 ± 0.012) were close to the original dataset (0.961 ± 0.004 accuracy, 0.962 ± 0.004 F1), showing minimal degradation. However, at higher editing levels (30–40%), performance slightly decreased (0.9442 ± 0.0142 accuracy, 0.9466 ± 0.0128 F1), indicating modest sensitivity to extensive paraphrasing. Interestingly, recall remained consistently high (0.987–0.988 ± 0.003), reflecting the model’s stability to detect positive cases. Calibration metrics (Brier/ECE) exhibited negligible variation across datasets, suggesting that human text edits particularly at moderate levels had limited influence on the reliability of RoBERTa’s confidence estimates.

**Table 9:** Comparison of results on the original dataset and human-edited version.

Dataset type	Accuracy (± CI)	Precision (± CI)	Recall (± CI)	F1 (± CI)	Brier (± CI)	ECE (± CI)
5–10% Human Edit	0.951 ± 0.014	0.920 ± 0.026	0.988 ± 0.003	0.953 ± 0.012	0.044 ± 0.012	0.491 ± 0.003
30–40% Human Edit	0.9442 ± 0.0142	0.9094 ± 0.0235	0.9871 ± 0.0024	0.9466 ± 0.0128	0.0501 ± 0.0118	0.4896 ± 0.0032
Actual Data	0.961 ± 0.004	0.945 ± 0.007	0.979 ± 0.004	0.962 ± 0.004	0.034 ± 0.003	0.492 ± 0.010

**Fig. 6:**
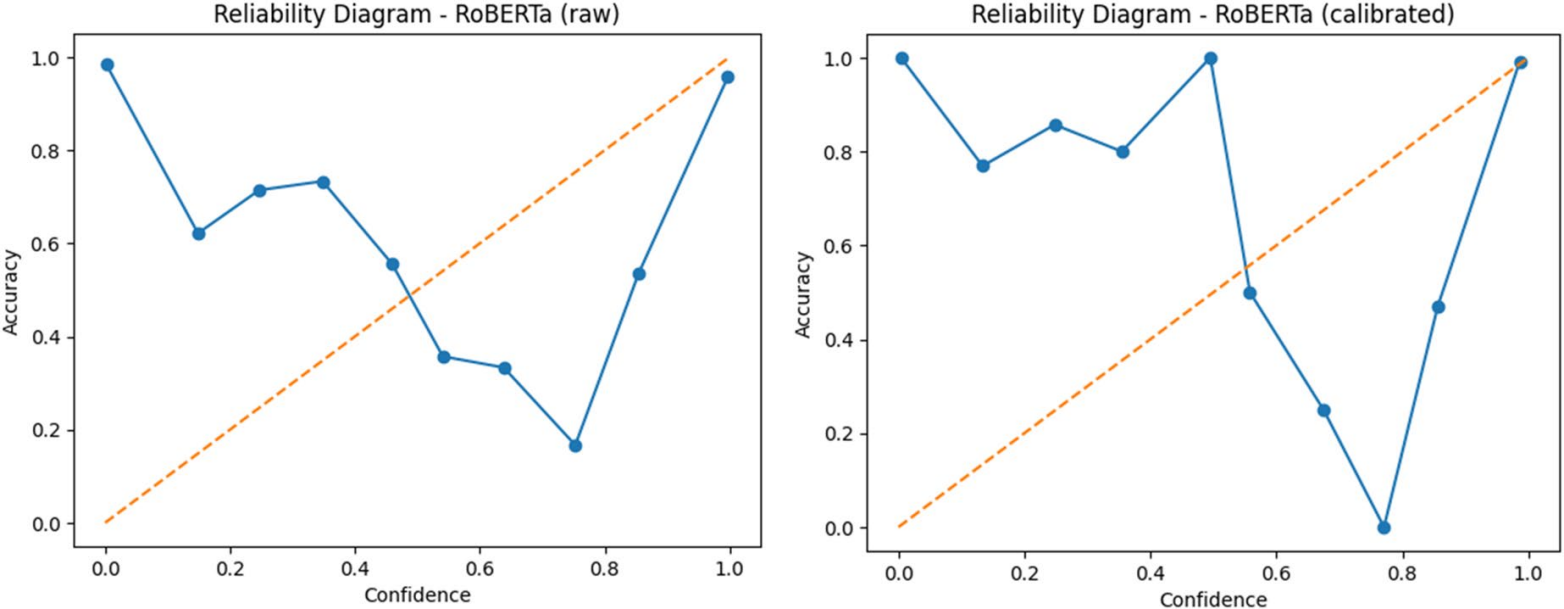
Reliability diagram of Roberta Model before calibration (left) and after calibration (right).

To assess the reliability of RoBERTa’s confidence estimates, temperature scaling was employed as a post-hoc calibration technique. The fitted temperature value was 1.476, which adjusted the model’s softmax outputs to better align predicted probabilities with actual outcomes. Before calibration, the Expected Calibration Error was approximately 0.4923, indicating substantial overconfidence. Temperature scaling effectively reduced miscalibration, improving the reliability of probability outputs. Figures 6 presents the reliability diagrams before and after calibration, respectively. The diagonal orange line represents perfect calibration, while deviations from this line reflect over- or under-confidence. As seen, calibration improves the model’s reliability across most confidence bins.

In addition, threshold tuning was performed to prioritize precision for high-stakes predictions. The optimal threshold achieving ≥ 90% precision was t = 0.957, resulting in precision = 0.963 and recall = 0.963. These adjustments enhance the interpretability and trustworthiness of the model’s outputs in practical applications.

To confirm whether observed performance differences between transformer models were statistically significant, McNemar’s test was conducted with Holm correction for multiple comparisons. The results (Table 10) revealed significant differences between all model pairs (*p* < 0.05). Specifically, XLM-RoBERTa vs. RoBERTa (*p* = 0.0195) and BERT vs. RoBERTa (*p* = 2.99 × 10⁻⁶) showed statistically reliable improvements in favor of RoBERTa. Although the effect sizes (Cohen’s g = 0.005–0.010) were small, they support the conclusion that RoBERTa’s performance advantages are consistent and not due to chance.

**Table 10:** Statistical comparison of top three transformer models using mcnemar’s Test, Holm Correction, and effect Sizes.

Model Pair	*p*-value	Holm-corrected *p*	Cohen’s g	ΔF1	Significant
XLM-RoBERTa vs. BERT	0.0184	0.0368	0.0056	+ 0.0051	✅
XLM-RoBERTa vs. RoBERTa	0.0195	0.0368	0.0050	− 0.0046	✅
BERT vs. RoBERTa	2.99 × 10⁻⁶	8.99 × 10⁻⁶	0.0106	− 0.0097	✅

In addition to accuracy metrics, inference efficiency was assessed through latency and throughput measurements (Table 11). RoBERTa achieved a balanced trade-off between speed and accuracy, with an average latency of 0.2935s per prediction and throughput of 68.1 texts/sec. XLM-RoBERTa demonstrated the highest throughput (69.1 texts/sec), while BERT was comparatively slower (63.2 texts/sec). These findings indicate that RoBERTa offers an optimal balance of computational cost and predictive reliability.

**Table 11:** Latency and throughput benchmarks for transformer models.

Model	Avg latency (s/prediction)	Throughput (texts/s)
XLM-RoBERTa	0.2893	69.1
BERT	0.3163	63.2
RoBERTa	0.2935	68.1

To assess potential for model compression, a global unstructured pruning experiment (20%) was conducted on RoBERTa. The pruned model maintained similar predictive behavior on a small validation sample, demonstrating the feasibility of parameter reduction without significant accuracy loss. This aligns with sustainability-oriented objectives by reducing computational demands while preserving interpretability.

A fine-grained error analysis was performed to evaluate RoBERTa’s robustness across text length categories. Results presented in Table 12 indicate that performance remained consistently high across all bins, with perfect scores for very short, short, and long inputs (F1 = 1.000) and only a minor drop for medium-length samples (F1 = 0.952). This suggests that the model generalizes effectively across varying input complexities and message lengths.

**Table 12:** RoBERTa performance across text-length bins.

Length Bin	*N*	Accuracy	Precision	Recall	F1-score
Very Short	3	1.000	1.000	1.000	1.000
Short	11	1.000	1.000	1.000	1.000
Medium	40	0.950	0.952	0.952	0.952
Long	46	1.000	1.000	1.000	1.000

### Explanations results

**Fig. 7:**
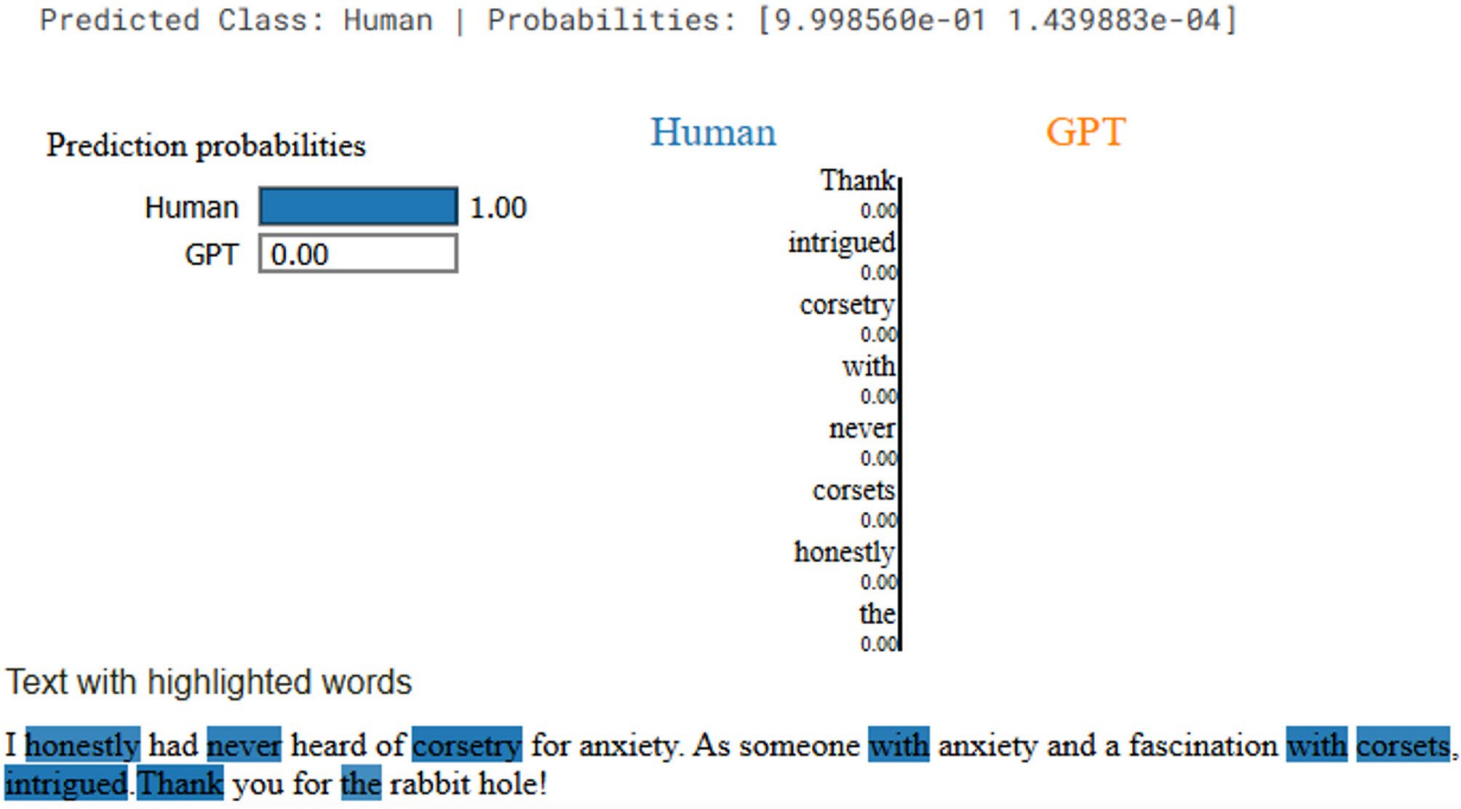
Explanation result of LIME for human generated text.

To enhance model transparency, LIME and SHAP was applied to the RoBERTa model predictions. LIME explains individual predictions by perturbing input text and approximating the model’s decision boundary with a simpler, interpretable model. As shown in Fig. 7, words such as *“honestly*,*” “never*,*” “corsetry*,*”* and *“intrigued”* were highlighted as strong contributors toward the Human class. The color intensity represents each token’s influence on the classification, helping to understand which linguistic features RoBERTa used in making its decision.

**Fig. 8:**
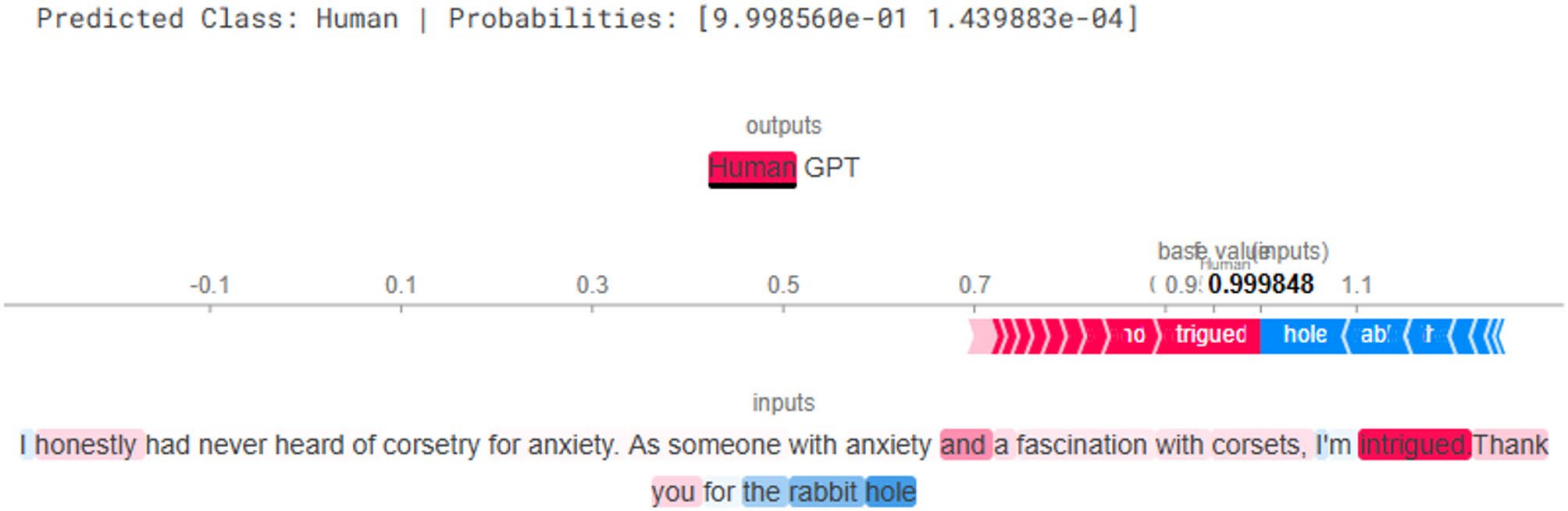
Explanation result of SHAP for human generated text.

Additionally, SHapley Additive Explanations provided a more theoretically grounded interpretation. SHAP assigns Shapley values to each token, indicating their positive or negative contributions to the output probability. In Fig. 8, red-colored tokens such as *“intrigued”* push the prediction toward the Human class, while blue tokens like *“the rabbit hole”* slightly pull it in the opposite direction. SHAP ensures that the contributions sum up to the predicted probability, offering a globally consistent and fair explanation of feature importance.

**Fig. 9:**
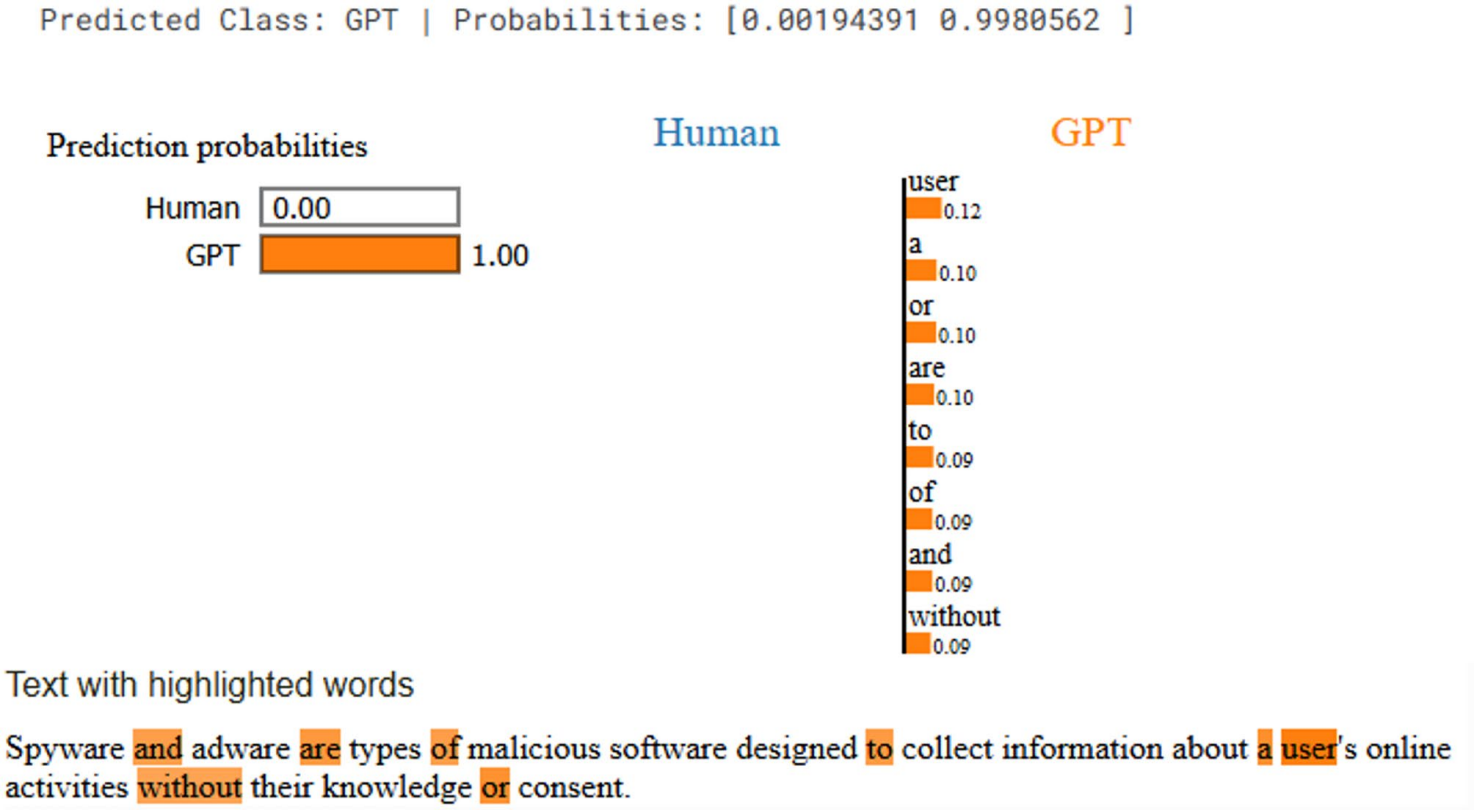
Explanation result of LIME for GPT generated text.

In the Fig. 9 focused on the LIME explanation shows that the model classified the input text as GPT with 100% probability, leaving no chance for Human. The highlighted words such as *“and*,*” “are*,*” “of*,*” “to*,*” “without*,*”* and *“user”* contributed most to the GPT prediction. These are mostly function words and connectors, which LIME suggests are strong signals of GPT-generated writing. In other words, the model associates GPT text with structured sentence flow and frequent use of linking terms, rather than with domain-specific keywords.

**Fig. 10:**
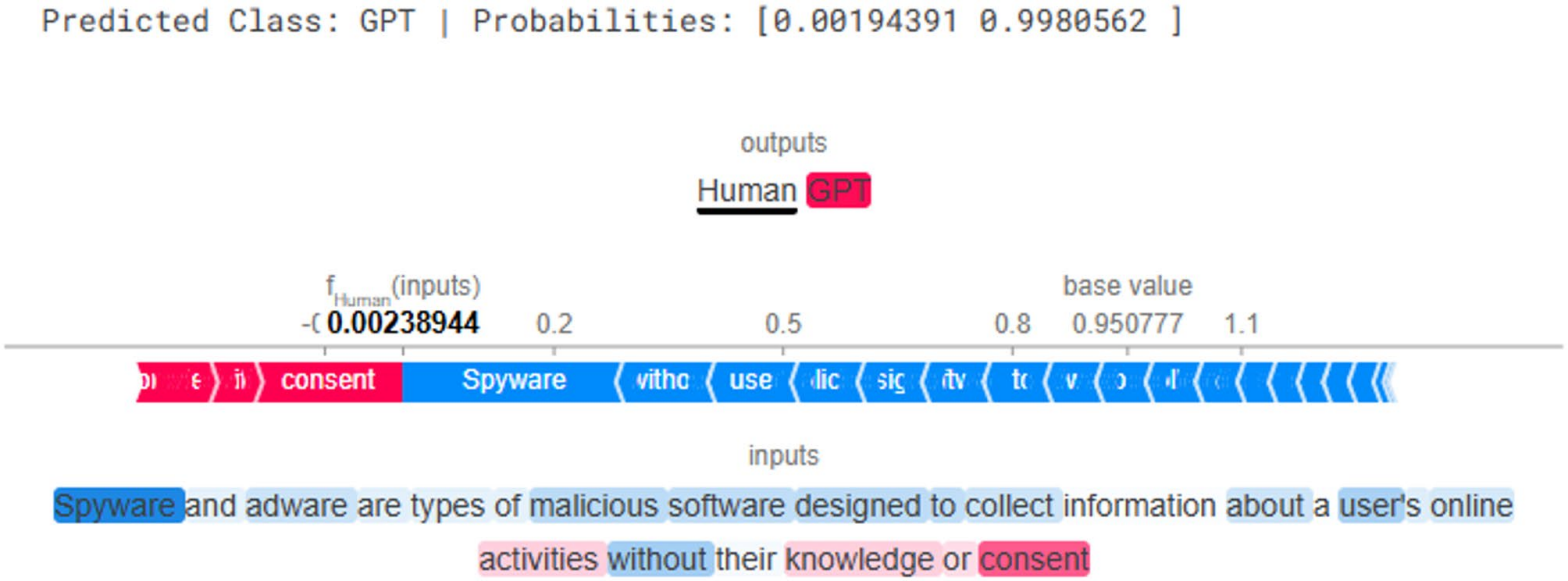
Explanation result of SHAP for GPT generated text.

The SHAP explanation (Fig. 10) also predicted the text as GPT with a probability of 0.9980. Unlike LIME, SHAP distinguishes between words pushing the prediction towards Human (blue) and GPT (red). Terms such as “Spyware,” “designed,” and “collect” leaned towards Human classification, as they resemble natural human writing and technical terminology. However, words like “without” and “consent” strongly pushed the decision towards GPT, highlighting how formal connectors and rigid phrasing are characteristic of machine-generated text.

In short, LIME provides a quick and visually intuitive understanding of which words influence RoBERTa’s predictions, making it ideal for fast debugging and local interpretability. SHAP, on the other hand, offers a more precise and mathematically consistent explanation by fairly distributing contributions among all tokens. While LIME is computationally lighter and easier to implement, SHAP is preferred when a deeper, globally consistent interpretation is required, especially in research or high-stakes decision-making scenarios.

**Fig. 11:**
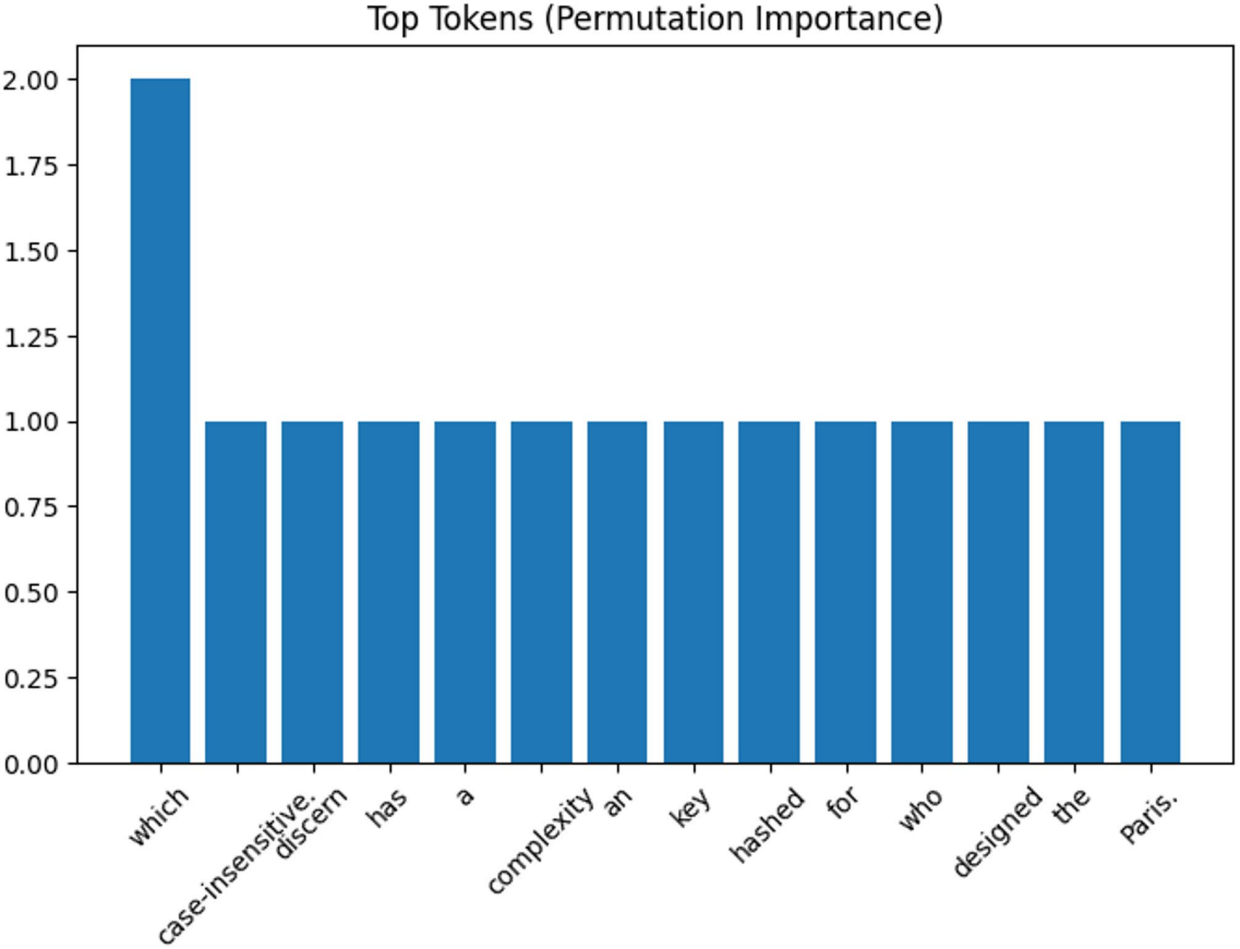
Top tokens using permutation feature importance plot.

In addition to local explainability, which focuses on understanding individual predictions, global explainability provides a broader view of the model’s behavior across the entire dataset. As shown in the Permutation Feature Importance (PFI) plot (Fig. 11), the token *“which”* stands out with the highest importance score of 2.0, indicating it has the greatest impact on model predictions when perturbed. Other tokens such as *“case-insensitive.”*, *“discern”*, *“complexity”*, and *“Paris.”* have lower but consistent importance values of 1.0, suggesting they also contribute meaningfully to the model’s overall decision-making. The baseline accuracy of 0.975 further supports the model’s robustness. To ensure global stability, agreement metrics across multiple runs or random seeds can be incorporated, confirming that the importance rankings are not sensitive to small variations in training.

## Discussion and implications

### Discussion of results

The results of this study underscore the superior capability of transformer-based architectures in distinguishing between human-generated and GPT-generated text. As detailed in Sect. 4.3, traditional machine learning models such as Random Forest, SVM, and Decision Trees attained moderate performance, with accuracies ranging from 80 to 85%. Sequential architectures like GRU and BiLSTM, leveraging FastText and GloVe embeddings, provided a modest improvement, reaching around 91–92%. However, transformer-based models—including BERT, DistilBERT, RoBERTa, and XLM-RoBERTa—demonstrated a significant leap in accuracy and robustness. Among them, RoBERTa achieved the best performance, with an overall accuracy of 96.1%, F1-score of 0.962, and a notably low Brier score of 0.040, reflecting strong calibration and generalization.

**Fig. 12:**
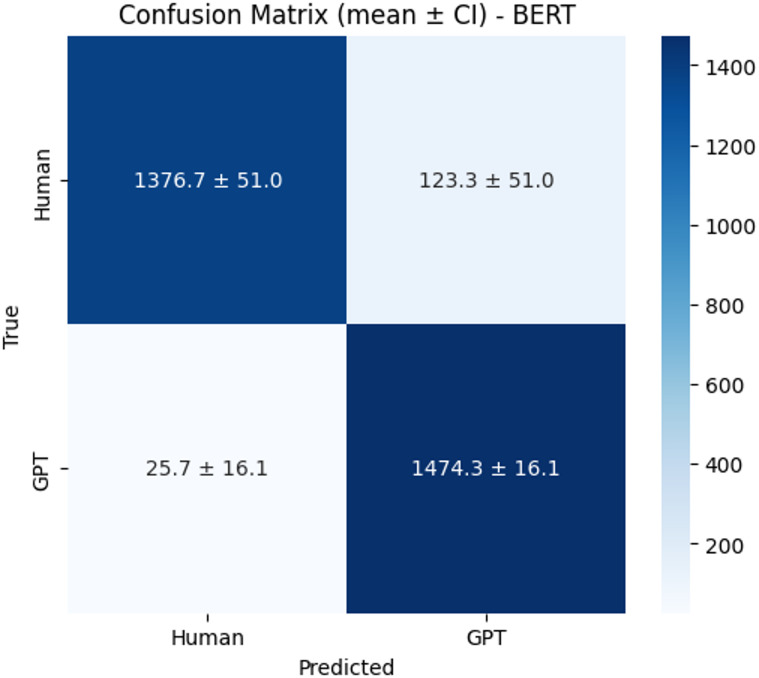
Confusion matrix of BERT.

**Fig. 13:**
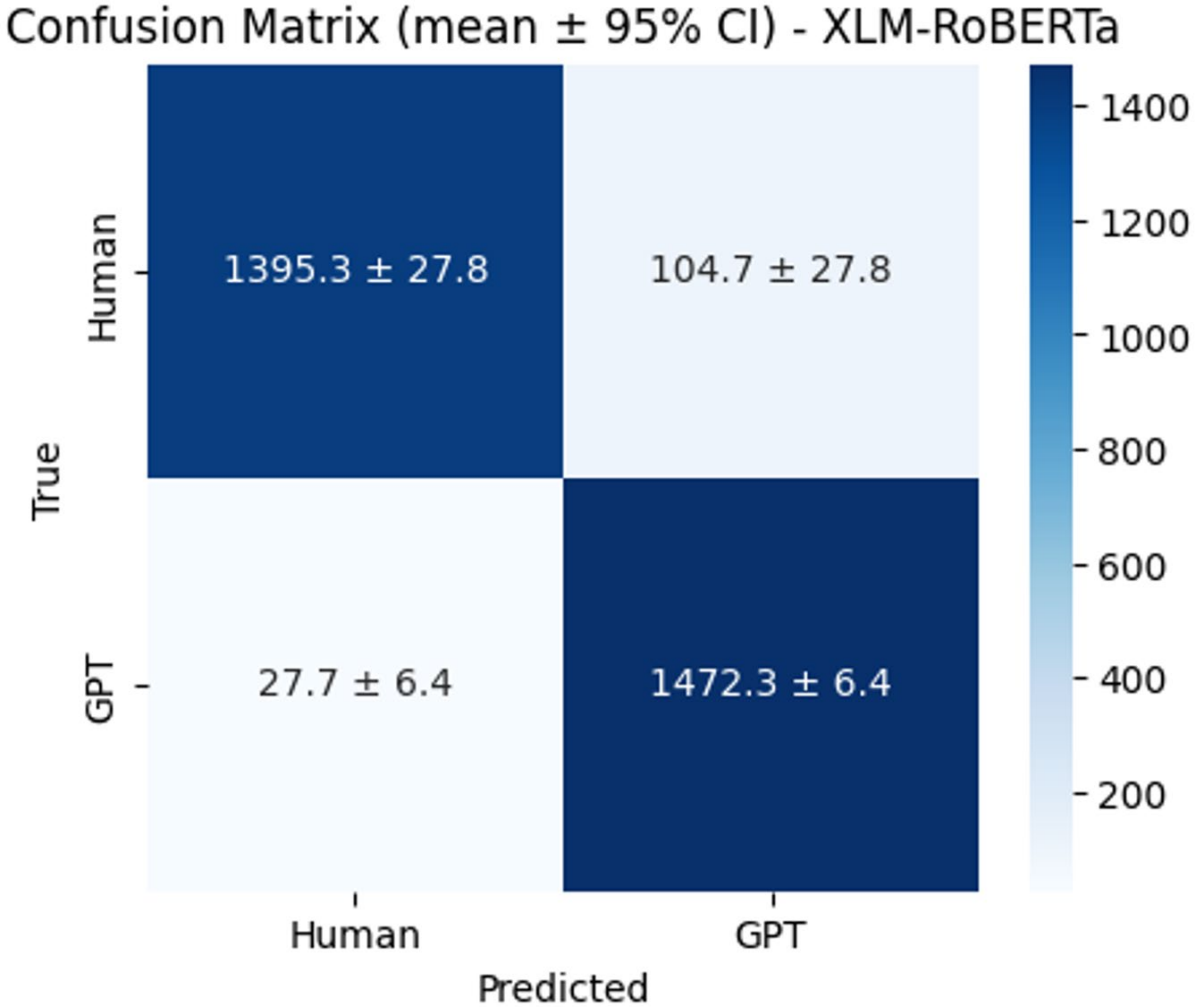
Confusion matrix of XLM-Roberta.

**Fig. 14:**
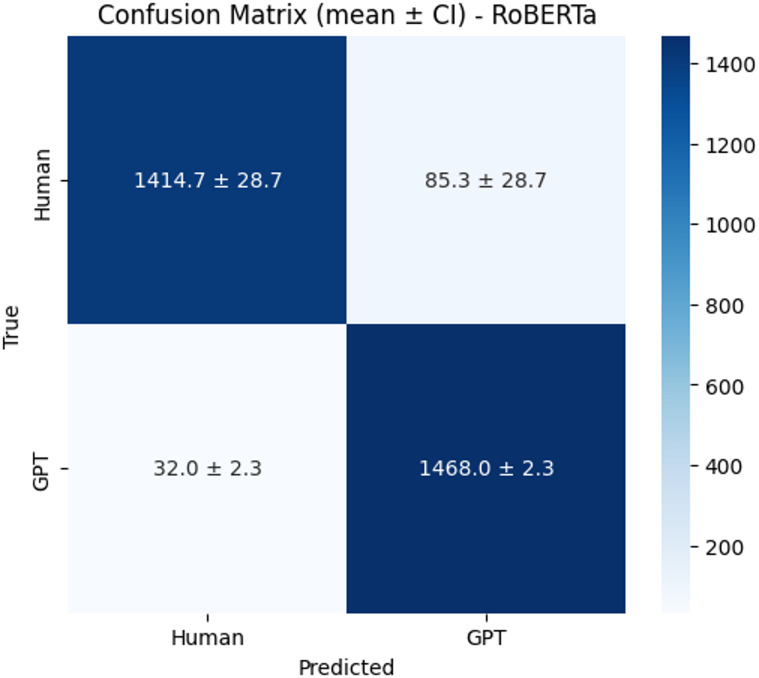
Confusion matrix of RoBERTa.

The confusion matrices (Figs. 12, 13 and 14) further highlight model distinctions. BERT correctly identified an average of 1,376.7 human and 1,474.3 GPT texts, though it tended to misclassify human texts as GPT more often than the reverse. XLM-RoBERTa showed similar human-text classification but slightly weaker GPT precision. In contrast, RoBERTa delivered the strongest and most balanced results, correctly classifying 1,414.7 human and 1,468.0 GPT samples while minimizing false predictions. These findings confirm that RoBERTa not only achieved the best quantitative performance but also maintained robust precision–recall balance with narrow confidence intervals, indicating stable and reliable performance across runs.

Interpretability analysis using LIME and SHAP provided valuable insights into the linguistic patterns influencing model decisions. LIME exposed the overreliance of GPT-generated text on structural connectors and filler terms (e.g., “and,” “of,” “to”), in contrast to the more content-rich and contextually grounded lexicon of human authors (e.g., “honestly,” “implement,” “intrigued”). SHAP results corroborated these findings by quantifying token-level contributions, confirming that redundancy and uniform phrasing are strong indicators of AI authorship. This interpretive transparency enhances trust in transformer models and aligns with current calls for explainable and auditable NLP systems.

In comparison to prior studies (e.g^15,20,22,23^. , , the proposed framework achieved competitive or superior performance while addressing key gaps of balanced data and explainability as shown in Table 13. Previous works often relied on smaller or outdated datasets and lacked interpretability layers. This study’s balanced dataset of 20,000 samples equally representing ChatGPT-3.5 and GPT-4 texts combined with XAI integration (LIME and SHAP), marks a significant step toward responsible and interpretable AI detection. Despite RoBERTa’s strong performance, computational intensity remains a practical constraint, suggesting potential for optimized variants such as DistilBERT^39^, which maintains accuracy while reducing computational cost.

**Table 13:** Comparison with previous studies.

References	Year	Dataset	No. of records	Algorithm	Results (accuracy)
^15^	2020	AI and Human Classification	10,000	SVM, KNN, NB, DT Logistic regression	77%
^21^	2023	AI and human medical text	10,000	BERT-based model	95%
^22^	2023	AI and Human Classification	10,000	TSA (LSTMRNN)	93.17%
^23^	2024	Reddit Dataset	6000	XGBoost algorithm, Random Forests and Deep Neural Networks	96%
^20^	2023	OpenGPTText	30,000	RoBERTa-Sentinel, T5-Sentinel	97%
^38^	2024	AI and human generated text	3000 data points	BERT, SVM, XGB	93%
Purposed Study	2025	GPT4 and 3.5 Balnce dataset	20,000	Machine Learning models like FastText + RF, GloVe + DT, Deep Learning models like FastText + RNN, GloVe + GRU and Transfer Learning Models like Roberta, DeRoberta etc. Explaination using LIME and SHAP	96.1%

### Practical implications

From an applied NLP perspective, the findings of this study carry important implications for the deployment, governance, and ethical oversight of AI-generated text detection systems. RoBERTa’s superior calibration and precision make it particularly suitable for high-stakes domains, such as academic integrity verification, journalism, corporate communication auditing, and content authenticity monitoring. However, the study emphasizes that model performance alone is insufficient without operational safeguards.

To mitigate the ethical risks of false positives particularly misclassifying human-authored text as GPT-generated a precision-preferred operational policy is recommended. Systems should enforce a minimum GPT-classification precision of 0.98, using probabilistic thresholds (e.g., P(GPT) ≥ 0.95) to ensure high-confidence labeling. Texts within ambiguous probability bands (0.05 < P(GPT) < 0.95) should be escalated to human-in-the-loop review, preserving fairness and accountability. This tiered approach harmonizes automation with human judgment, reducing potential reputational or academic harm.

Moreover, the integration of Explainable AI tools such as LIME and SHAP in production environments enhances transparency and auditability, allowing end-users and reviewers to trace and interpret model reasoning. This is particularly relevant in compliance-driven sectors that require justification of algorithmic decisions under data governance frameworks.

Future implementations can benefit from model distillation and edge-based optimization, enabling scalable deployment without sacrificing interpretability^42–44^. Expanding this framework to other domains such as creative writing, policy drafting, and technical documentation can provide broader validation. The inclusion of newer LLMs such as GPT-4, LLaMA and metadata-annotated datasets will further enable diagnostic analysis of contextual errors and model biases.

Ultimately, this study not only contributes a high-performing detection model but also advances a governance-oriented framework emphasizing ethical deployment, calibrated decision-making, and transparency. This aligns with contemporary standards in Responsible AI, reinforcing public trust in automated content verification systems and setting a precedent for the ethical use of NLP technologies in an increasingly AI-mediated communication landscape.

## Conclusion

This study presented a comprehensive investigation into the detection of AI-generated text, focusing on distinguishing between human-authored and ChatGPT-generated content through a multi-layered experimental framework encompassing traditional machine learning, recurrent deep learning, and transformer-based models. The results clearly demonstrated the superiority of transformer architectures over both classical and sequential models, with RoBERTa achieving the highest accuracy (96.1%), supported by strong precision, recall, and F1-scores. These findings affirm the effectiveness of contextualized embeddings and self-attention mechanisms in capturing subtle linguistic and stylistic differences between human and AI text.

Beyond accuracy, the study emphasized reliability, interpretability, and sustainability the three key pillars for trustworthy AI deployment. Through temperature scaling, RoBERTa’s confidence estimates were successfully calibrated, reducing overconfidence and aligning predicted probabilities with actual outcomes. Threshold tuning enabled precision-prioritized predictions, enhancing model trustworthiness for high-stakes applications such as academic integrity verification, misinformation detection, and authorship authentication. Moreover, statistical testing using McNemar’s test confirmed that RoBERTa’s performance improvements were statistically significant, not coincidental. The pruning experiment demonstrated that model compression could be achieved without substantial loss of predictive accuracy, contributing to sustainable and efficient AI usage. Furthermore, LIME and SHAP explainability methods provided transparent insight into model behavior, revealing that AI-generated texts tend to rely on structured phrasing and frequent connectors, whereas human-authored texts exhibit expressive variability and domain-specific richness. The fine-grained error analysis further showed that RoBERTa maintained consistent robustness across varying text lengths, validating its generalization capability across diverse input complexities.

Overall, the study concludes that RoBERTa offers the most reliable, interpretable, and computationally balanced solution for distinguishing AI-generated text from human-authored content. However, future work should expand the dataset to include outputs from emerging large language models (e.g., Gemini, Claude, Mistral) and explore hybrid architectures. Additionally, incorporating genre- and topic-level error analyses will further improve domain adaptability and threshold calibration for real-world deployment. By integrating performance excellence with interpretability and ethical awareness, this research contributes to the growing field of AI transparency and content authenticity verification, setting a foundation for sustainable and explainable NLP systems in the age of generative AI.

## Data Availability

The datasets used in this study are publicly available from Kaggle and GitHub repositories. The original sources are cited in Table 1 of the manuscript, enabling readers to directly access the repositories for details regarding collection context and availability. While these platforms provide open access under their respective terms of use, the datasets do not explicitly specify licensing or formal data cards. Whereas the code used for this study is openly available for reproducibility at [https://github.com/shamylafirdoos/Gpt-vs-Human-Text-Classification](https:/github.com/shamylafirdoos/Gpt-vs-Human-Text-Classification).

## References

[1] Luo, Z., Yang, Z., Xu, Z., Yang, W. & Du, X. LLM4SR: A Survey on Large Language Models for Scientific Research, Jan. Accessed: Jul. 12, 2025. [Online]. (2025). Available: http://arxiv.org/abs/2501.04306

[2] Naveed, H. et al. A comprehensive overview of large Language models. *Int. J. Multidisciplinary Res.* **7** (1). 10.36948/ijfmr.2025.v07i01.34609 (Jul. 2023).

[3] Minaee, S. et al. Feb., Large Language Models: A Survey, Accessed: Jul. 12, 2025. [Online]. (2024). Available: https://arxiv.org/pdf/2402.06196

[4] Brown, T. B. et al. Language Models are Few-Shot Learners, *Adv Neural Inf Process Syst*, vol. 2020-December, May 2020, Accessed: Jul. 12, 2025. [Online]. Available: https://arxiv.org/pdf/2005.14165

[5] Prova, N., Detecting, A. I. & Generated Text Based on NLP and Machine Learning Approaches. Apr., Accessed: Jul. 12, 2025. [Online]. (2024). Available: https://arxiv.org/pdf/2404.10032

[6] Chandana, I., Reshma, O. M., Sree, N. G., Reddy, B. J. & Shareefunnisa, S. Detecting AI Generated Text, in *2nd World Conference on Communication and Computing, WCONF 2024*, Institute of Electrical and Electronics Engineers Inc., 2024., Institute of Electrical and Electronics Engineers Inc., 2024. (2024). 10.1109/WCONF61366.2024.10692028

[7] Jadhwani, S., Jain, S. & Doshi, P. Detecting AI generated content in short form text. *Jan*10.21203/RS.3.RS-5331372/V1 (2025).

[8] Rosenfeld, R. Two decdes of statistical language modeling where do we go form here? Where do we go from here? in *Proceedings of the IEEE*, Institute of Electrical and Electronics Engineers Inc., pp. 1270–1275. (2000). 10.1109/5.880083

[9] Song, Y. & Kingma, D. P. How to Train Your Energy-Based Models, Jan. Accessed: Jul. 12, 2025. [Online]. (2021). Available: https://arxiv.org/pdf/2101.03288

[10] Yin, Q., Han, C., Li, A., Liu, X. & Liu, Y. A review of research on Building energy consumption prediction models based on artificial neural networks. *Sustain. 2024*. **16, Page 7805, 16**, (17), 7805. 10.3390/SU16177805 (Sep. 2024).

[11] Hochreiter, S., Schmidhuber, J. & Memory, L. S. T. *Neural Comput*, vol. 9, no. 8, pp. 1735–1780, Nov. (1997). 10.1162/NECO.1997.9.8.1735 10.1162/neco.1997.9.8.1735 9377276

[12] Chung, J., Gulcehre, C., Cho, K. & Bengio, Y. Empirical Evaluation of Gated Recurrent Neural Networks on Sequence Modeling, Dec. Accessed: Jul. 12, 2025. [Online]. (2014). Available: https://arxiv.org/pdf/1412.3555

[13] Devlin, J., Chang, M. W., Lee, K. & Toutanova, K. BERT: Pre-training of Deep Bidirectional Transformers for Language Understanding, *Proceedings of the Conference of the North*, pp. 4171–4186, 2019, pp. 4171–4186, 2019, (2019). 10.18653/V1/N19-1423

[14] Sanh, V., Debut, L., Chaumond, J. & Wolf, T. DistilBERT, a distilled version of BERT: smaller, faster, cheaper and lighter, Oct. Accessed: Jul. 12, 2025. [Online]. (2019). Available: https://arxiv.org/pdf/1910.01108

[15] Islam, N. et al. Distinguishing Human Generated Text From ChatGPT Generated Text Using Machine Learning, May 2023, Accessed: Jul. 12, 2025. [Online]. Available: https://arxiv.org/pdf/2306.01761

[16] Ribeiro, M. T., Singh, S. & Guestrin, C. ‘Why Should I Trust You?’: Explaining the Predictions of Any Classifier, *NAACL-HLT –2016 Conference of the North American Chapter of the Association for Computational Linguistics: Human Language Technologies, Proceedings of the Demonstrations Session*, pp. 97–101, Feb. 2016, pp. 97–101, Feb. 2016, (2016). 10.18653/v1/n16-3020

[17] Ippolito, D., Duckworth, D., Callison-Burch, C. & Eck, D. Automatic Detection of Generated Text is Easiest when Humans are Fooled, *Proceedings of the Annual Meeting of the Association for Computational Linguistics*, pp. 1808–1822, Nov. (2019). 10.18653/v1/2020.acl-main.164

[18] Solaiman, I. et al. Aug., Release Strategies and the Social Impacts of Language Models, Accessed: Jul. 12, 2025. [Online]. (2019). Available: https://arxiv.org/pdf/1908.09203

[19] Jawahar, G., Abdul-Mageed, M. & Lakshmanan, L. V. S. Automatic Detection of Machine Generated Text: A Critical Survey, in *COLING –28th International Conference on Computational Linguistics, Proceedings of the Conference*, Association for Computational Linguistics (ACL), 2020, pp. 2296–2309., Association for Computational Linguistics (ACL), 2020, pp. 2296–2309. (2020). 10.18653/V1/2020.COLING-MAIN.208

[20] Chen, Y. et al. GPT-Sentinel: Distinguishing Human and ChatGPT Generated Content, May Accessed: Jul. 12, 2025. [Online]. (2023). Available: https://arxiv.org/pdf/2305.07969

[21] Liao, W. et al. Differentiate ChatGPT-generated and Human-written medical texts. *JMIR Med. Educ.* **9** (1). 10.2196/48904 (Apr. 2023). 10.2196/48904 PMC10784984 38153785

[22] Katib, I., Assiri, F. Y., Abdushkour, H. A., Hamed, D. & Ragab, M. Differentiating Chat Generative Pretrained Transformer from Humans: Detecting ChatGPT-Generated Text and Human Text Using Machine Learning, *Mathematics*, vol. 11, no. 15, pp. 1–19, 2023, Accessed: Jul. 12, 2025. [Online]. (2023). Available: https://ideas.repec.org/a/gam/jmathe/v11yi15p3400-d1210228.html.

[23] Qazi, Z., Shiao, W. & Papalexakis, E. E. GPT-generated Text Detection: Benchmark Dataset and Tensor-based Detection Method, *Companion Proceedings of the ACM Web Conference (WWW ’24 Companion), May 13â•fi17, 2024, Singapore, Singapore*, vol. 1, Mar. 2024, vol. 1, Mar. 2024, (2024). 10.1145/3589335.3651513

[24] Qazi, Z., Shiao, W. & Papalexakis, E. E. GPT-generated Text Detection: Benchmark Dataset and Tensor-based Detection Method, *Companion Proceedings of the ACM Web Conference (WWW ’24 Companion), May 13â•fi17, 2024, Singapore, Singapore*, vol. 1, 2024, vol. 1, 2024, (2024). 10.1145/3589335.3651513

[25] Mahdi ChatGPT Classification Dataset. Accessed: Sep. 17, 2025. [Online]. Available: https://www.kaggle.com/datasets/mahdimaktabdar/chatgpt-classification-dataset?select=sentence_level_data.csv

[26] Devastator, T. All GPT-4 Conversations. Accessed: Sep. 17, 2025. [Online]. Available: https://www.kaggle.com/datasets/thedevastator/all-gpt-4-synthetic-chat-datasets

[27] Goldberg, Y. et al. word2vec Explained: deriving Mikolov et alFeb. ’s negative-sampling word-embedding method, Accessed: Jul. 13, 2025. [Online]. (2014). Available: https://arxiv.org/pdf/1402.3722

[28] Pennington, J., Socher, R. & Manning, C. D. GloVe: Global vectors for word representation, in *EMNLP –2014 Conference on Empirical Methods in Natural Language Processing, Proceedings of the Conference*, Association for Computational Linguistics (ACL), 2014, pp. 1532–1543., Association for Computational Linguistics (ACL), 2014, pp. 1532–1543. (2014). 10.3115/V1/D14-1162

[29] Santos, F. A. O., Macedo, H. T., Dias Bispo, T. & Zanchettin, C. Morphological Skip-Gram: Using morphological knowledge to improve word representation, Jul. Accessed: Jul. 02, 2025. [Online]. (2020). Available: https://arxiv.org/pdf/2007.10055

[30] Afif, M. H., Hedar, A. R., Hamid, T. H. A. & Mahdy, Y. B. Support vector machines with weighted powered kernels for data classification, in Communications in Computer and Information Science, Springer, 369–378. doi: 10.1007/978-3-642-35326-0_37. (2012).

[31] Hansen, J. Diabetic risk prognosis with tree ensembles integrating feature attribution methods. *Evol. Intell.* **17** (1), 419–428. 10.1007/S12065-021-00663-1 (Feb. 2024).

[32] Mienye, I. D., Swart, T. G. & Obaido, G. Recurrent neural networks: A comprehensive review of Architectures, Variants, and applications. *Inform. 2024*. **15** (9), 517. 10.3390/INFO15090517 (Aug. 2024). Page 517.

[33] Tarigan, G. A., Hermawan, E. & Girsang, A. S. Parallelization of LSTM-GRU Architectures for Multivariate Prediction of Stock Prices, in *Proceedings of International Conference on Information Management and Technology, ICIMTech 2024*, Institute of Electrical and Electronics Engineers Inc., 2024, pp. 311–315., Institute of Electrical and Electronics Engineers Inc., 2024, pp. 311–315. (2024). 10.1109/ICIMTECH63123.2024.10780885

[34] Wang, J. et al. Utilizing BERT for information retrieval: Survey, Applications, Resources, and challenges. *ACM Comput. Surv.* **56** (7). 10.1145/3648471 (Feb. 2024).

[35] Shu, X. BERT and RoBERTa for sarcasm detection: optimizing performance through advanced Fine-tuning. *Appl. Comput. Eng.* **97** (1), 1–11. 10.54254/2755-2721/97/20241354 (Nov. 2024).

[36] Chai, Y., Liang, Y. & Duan, N. Cross-Lingual Ability of Multilingual Masked Language Models: A Study of Language Structure, in *Proceedings of the Annual Meeting of the Association for Computational Linguistics*, Association for Computational Linguistics (ACL), Mar. pp. 4702–4712. (2022). 10.18653/v1/2022.acl-long.322

[37] Sinha, S., Chen, H., Sekhon, A., Ji, Y. & Qi, Y. Perturbing Inputs for Fragile Interpretations in Deep Natural Language Processing, in *BlackboxNLP 2021 - Proceedings of the 4th BlackboxNLP Workshop on Analyzing and Interpreting Neural Networks for NLP*, Association for Computational Linguistics (ACL), Aug. pp. 420–434. (2021). 10.18653/v1/2021.blackboxnlp-1.33

[38] DiPietro, R. & Hager, G. D. Deep learning: RNNs and LSTM, *Handbook of Medical Image Computing and Computer Assisted Intervention*, pp. 503–519, Jan. (2019). 10.1016/B978-0-12-816176-0.00026-0

[39] Hinton, G., Vinyals, O. & Dean, J. Distilling the Knowledge in a Neural Network, Mar. Accessed: Jul. 12, 2025. [Online]. (2015). Available: https://arxiv.org/pdf/1503.02531

[40] Bhatnagar, S. et al. Feb., The Malicious Use of Artificial Intelligence: Forecasting, Prevention, and Mitigation, Accessed: Jul. 12, 2025. [Online]. (2018). Available: https://arxiv.org/pdf/1802.07228

[41] Lundberg, S. M. & Lee, S. I. A Unified Approach to Interpreting Model Predictions, *Adv Neural Inf Process Syst*, vol. 2017-December, pp. 4766–4775, May 2017, Accessed: Jul. 12, 2025. [Online]. Available: https://arxiv.org/pdf/1705.07874

[42] Radford, A. et al. Language Models are Unsupervised Multitask Learners. [Online]. Available: https://github.com/codelucas/newspaper

[43] He, H. & Garcia, E. A. Learning from imbalanced data, *IEEE Trans Knowl Data Eng*, vol. 21, no. 9, pp. 1263–1284, Sep. (2009). 10.1109/TKDE.2008.239

[44] Shi, W., Cao, J., Zhang, Q., Li, Y. & Xu, L. Edge computing: vision and challenges. *IEEE Internet Things J.* **3** (5), 637–646. 10.1109/JIOT.2016.2579198 (Oct. 2016).

